# Breastfeeding during infancy and neurocognitive function in adolescence: 16-year follow-up of the PROBIT cluster-randomized trial

**DOI:** 10.1371/journal.pmed.1002554

**Published:** 2018-04-20

**Authors:** Seungmi Yang, Richard M. Martin, Emily Oken, Mikhail Hameza, Glen Doniger, Shimon Amit, Rita Patel, Jennifer Thompson, Sheryl L. Rifas-Shiman, Konstantin Vilchuck, Natalia Bogdanovich, Michael S. Kramer

**Affiliations:** 1 Department of Epidemiology, Biostatistics and Occupational Health, Faculty of Medicine, McGill University, Montreal, Quebec, Canada; 2 School of Social and Community Medicine, University of Bristol, Bristol, United Kingdom; 3 University Hospitals Bristol NHS Foundation Trust National Institute for Health Research Bristol Nutrition Biomedical Research Unit, University of Bristol, Bristol, United Kingdom; 4 Department of Population Medicine, Harvard Medical School and Harvard Pilgrim Health Care Institute, Boston, Massachusetts, United States of America; 5 National Research and Applied Medicine Mother and Child Centre, Minsk, Republic of Belarus; 6 NeuroTrax Corporation, Medina, New York, United States of America; 7 Department of Pediatrics, Faculty of Medicine, McGill University, Montreal, Quebec, Canada; London School of Hygiene & Tropical Medicine, UNITED KINGDOM

## Abstract

**Background:**

Evidence on the long-term effect of breastfeeding on neurocognitive development is based almost exclusively on observational studies. In the 16-year follow-up study of a large, cluster-randomized trial of a breastfeeding promotion intervention, we evaluated the long-term persistence of the neurocognitive benefits of the breastfeeding promotion intervention previously observed at early school age.

**Methods and findings:**

A total of 13,557 participants (79.5% of the 17,046 randomized) of the Promotion of Breastfeeding Intervention Trial (PROBIT) were followed up at age 16 from September 2012 to July 2015. At the follow-up, neurocognitive function was assessed in 7 verbal and nonverbal cognitive domains using a computerized, self-administered test battery among 13,427 participants. Using an intention-to-treat (ITT) analysis as our prespecified primary analysis, we estimated cluster- and baseline characteristic-adjusted mean differences between the intervention (prolonged and exclusive breastfeeding promotion modelled on the Baby-Friendly Hospital Initiative) and control (usual care) groups in 7 cognitive domains and a global cognitive score. In our prespecified secondary analysis, we estimated mean differences by instrumental variable (IV) analysis to account for noncompliance with the randomly assigned intervention and estimate causal effects of breastfeeding. The 16-year follow-up rates were similar in the intervention (79.7%) and control groups (79.3%), and baseline characteristics were comparable between the two. In the cluster-adjusted ITT analyses, children in the intervention group did not show statistically significant differences in the scores from children in the control group. Prespecified additional adjustment for baseline characteristics improved statistical precision and resulted in slightly higher scores among children in the intervention for verbal function (1.4 [95% CI 0.3–2.5]) and memory (1.2 [95% CI 0.01–2.4]). IV analysis showed that children who were exclusively breastfed for ≥3 (versus <3) months had a 3.5-point (95% CI 0.9–6.1) higher verbal function, but no differences were observed in other domains. While our computerized, self-administered cognitive testing reduced the cluster-level variability in the scores, it may have increased individual-level measurement errors in adolescents.

**Conclusions:**

We observed no benefit of a breastfeeding promotion intervention on overall neurocognitive function. The only beneficial effect was on verbal function at age 16. The higher verbal ability is consistent with results observed at early school age; however, the effect size was substantially smaller in adolescence.

**PROBIT trial registration:**

ClinicalTrials.gov NCT01561612

## Abstract 2

The Promotion of Breastfeeding Intervention Trial (PROBIT) trial in Belarus showed benefits to neurocognitive function in early child development. In this follow-up, Seungmi Yang and colleagues examine the same outcomes at 16 years.

## Author summary

**Why was this study done?:**

- Breastfeeding has shown beneficial, long-term effects on neurocognitive development in childhood through adolescence.
- The evidence is almost exclusively based on observational studies, which are prone to confounding.
- PROBIT, the largest randomized controlled trial on human lactation, has found higher IQ scores at age 6.5 years among children randomly assigned at birth into a breastfeeding promotion intervention group than those in routine practice, particularly for verbal IQ.

**What did the researchers do and find?:**

- We conducted a follow-up examination of the PROBIT participants at age 16 to examine whether the beneficial effects observed in early school age persisted in adolescence.
- A computerized, self-administered neurocognitive function test measured 7 different verbal and nonverbal cognitive domains, as well as global cognitive function.
- The intervention to promote prolonged and exclusive breastfeeding showed little evidence on beneficial effects on overall neurocognitive function at age 16, and a modest benefit was found for verbal function.

**What do these findings mean?:**

- Results of our findings at age 16 combined with results at age 6.5 years suggest that long-term effects of breastfeeding on neurocognitive development decrease in magnitude with advancing age, and the persistent benefit seems to be limited to verbal function.

## Introduction

Improved neurocognitive development has been reported as one of the long-term benefits of having been breastfed [1]. A recent meta-analysis of 17 observational studies reported that breastfeeding was associated with higher intelligence quotient (IQ) scores by an average of 3.4 (95% CI 2.3–4.6) points in children at ages 1–19 years overall, with differing effects by age groups (4.1 [95% CI 2.5–5.7] points in ages 1–9 years and 1.9 [95% CI 0.4–3.4] points in ages 10–19 years) [2]. A Brazilian birth cohort recently showed positive associations of breastfeeding not only with cognitive ability but also with income at age 30 years [3]. However, residual confounding by unmeasured maternal and family characteristics that affect both breastfeeding and child cognitive ability is an inherent limitation of observational studies. In a comparative study of two cohorts from the United Kingdom and Brazil, breastfeeding has been associated with higher IQ scores in both cohorts with different social patterning of breastfeeding, thus better accounting for residual confounding by socioeconomic factors [4]. On the other hand, studies comparing siblings within families—a study design that may also better control for confounding—have reported conflicting results [5–7]. Thus, despite attempts at improved control for confounding, results from observational studies are inconclusive about the causal relationship between breastfeeding and later neurocognitive function.

Two controlled trials have examined beneficial effects of breast milk or breastfeeding on neurocognitive development. One examined the effect of donor breast milk or nutrient-enriched “preterm” formula versus standard formula among 502 preterm infants. The investigators observed higher development scores in preterm formula- versus standard formula-fed children [8,9] but no differences between breast milk-fed and preterm formula-fed infants [10]. The other is the Promotion of Breastfeeding Intervention Trial (PROBIT), a cluster-randomized trial of breastfeeding promotion in the Republic of Belarus within which the present analysis is also based. In PROBIT, we previously reported that term infants randomized to the intervention had 7.5 points higher (95% CI 0.8–14.3) verbal IQ at age 6.5 years; 2.9 (95% CI −3.3–9.1) points higher performance IQ; and 5.9 (95% CI −1.0–12.8) points higher full-scale IQ [11]. However, this finding was limited by high within-site clustering (intraclass correlation coefficient [ICC] = 0.31) of cognitive scores, leading to imprecision with wide confidence intervals [12] in the effect estimates, and by potential bias due to nonblinding of study pediatricians who administered the cognitive test. In the present study, we examine whether the beneficial effects of breastfeeding we have observed at age 6.5 years persist at age 16 years in both verbal and nonverbal domains of neurocognitive function, using a computerized neurocognition assessment battery to overcome these limitations.

## Methods

### Study design and participants

**Fig 1:**
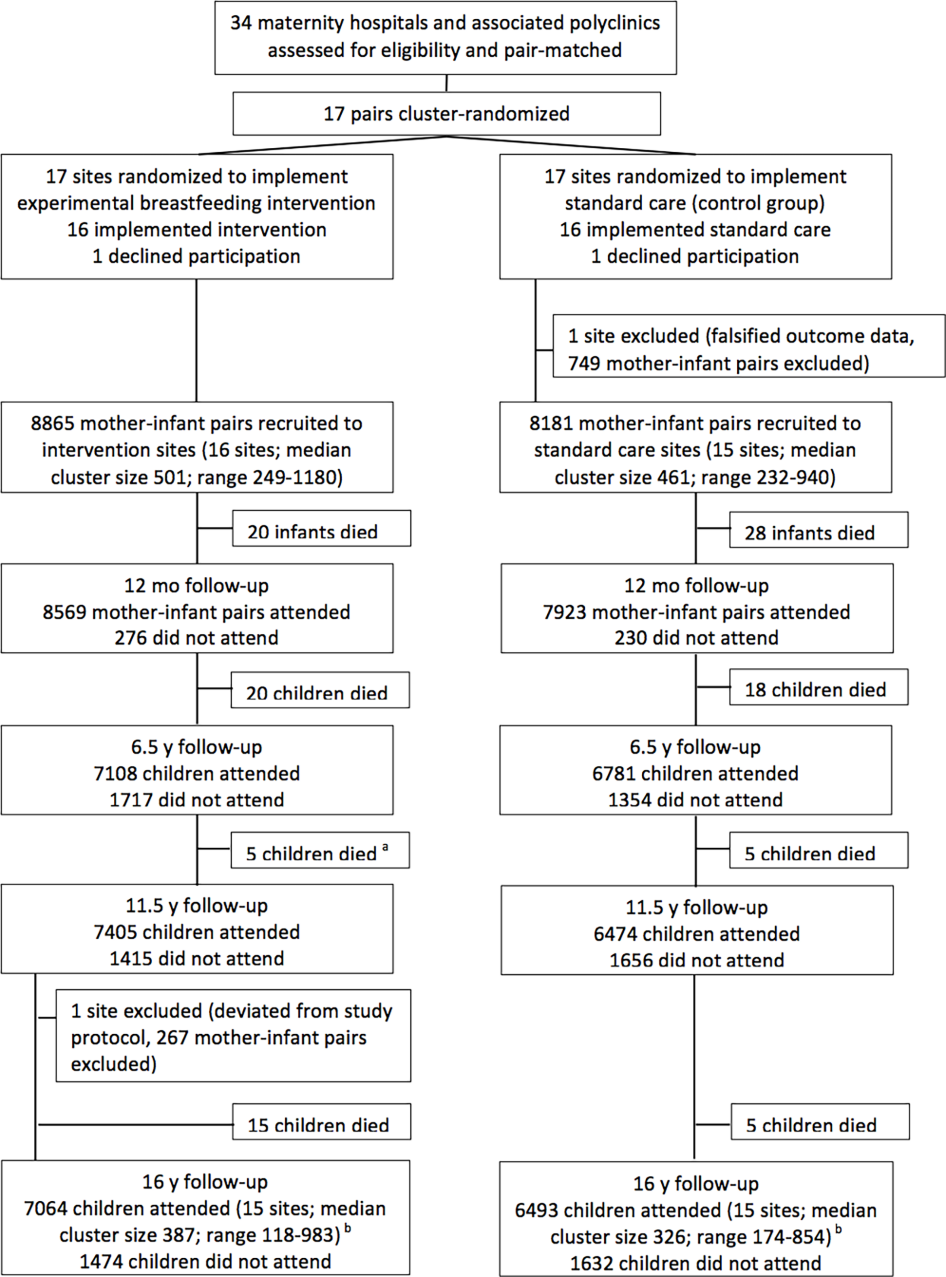
Flow diagram of clusters and participants of PROBIT recruitment follow-up phases at 12 mo, 6.5 y, 11.5 y, and 16 y. ^a^: During the 11.5-y follow-up, 6 deaths were reported in the intervention arm. Data-checking during the 16-y follow-up found one of these children had been incorrectly reported as deceased and data were amended. ^b^: Of the 13,557 seen at the 16-y follow-up, 12,072 were seen at both 11.5-y and 16-y follow-ups, 274 were not seen at either 6.5-y or 11.5-y follow-ups, 449 were seen at 6.5 y but not seen at 11.5 y, and 762 were seen at 11.5 y but not seen at 6.5 y. Of the 3,489 children randomized but not followed up at 16 y, 267 attended the excluded site, 116 died after randomization, 2,674 were lost to follow-up, and 432 were unable or unwilling to come for their clinic visit.

A full description of the trial design, experimental intervention, and participants in PROBIT has been published [13]. In brief, 31 maternity hospitals and their affiliated outpatient polyclinics (clusters) were paired according to 7 geographic regions and urban versus rural status, number of deliveries per year, and breastfeeding initiation rates at hospital discharge in order to balance the two randomized intervention groups. The clusters were randomized either to receive an intervention to promote both exclusive and prolonged breastfeeding (modeled on the Baby-Friendly Hospital Initiative developed by WHO and UNICEF) or to continue the maternity hospital and polyclinic standard practices in effect at the time of randomization, according to a double-randomization procedure. A two-digit random number from a random number table was first assigned to each pair, and within each pair, the cluster corresponding to the higher and lower numbers were assigned to groups A and B, respectively. At a public gathering of the PROBIT investigators, a coin flip determined that B clusters would receive the experimental intervention and A clusters would receive the control intervention. Fig 1 shows the overall study design from recruitment to the most recent follow-up. A total of 17,046 infants, who were healthy singletons born at ≥37 completed weeks of gestation with birth weight ≥2500 g and 5-minute Apgar score ≥5 and whose mothers expressed an intention to breastfeed on admission to the postpartum ward, were recruited during their postpartum stay between June 1996 and December 1997. Scheduled follow-up visits were made at 1, 2, 3, 6, 9, and 12 months, during which study pediatricians assessed infant feeding using standard questionnaires, which we validated against chart reviews. At research visits conducted at age 6.5 years, the pediatricians administered the Wechsler Abbreviated Scale of Intelligence (WASI) to assess verbal and performance IQ. The most recent follow-up at age 16 years included a total of 13,557 participants (79.5% of the original cohort) interviewed from September 2012 to July 2015. Trained pediatricians conducted the follow-up visits: 1 in each of 24 polyclinics, and 2 at the remaining 7 high-volume clinics. We ensured standardized data collection across pediatricians via tutoring, hands-on workshops, and ongoing data monitoring [14]. Data from one polyclinic (*n* = 169) were excluded because of major deviations from the study protocol.

The 16-year follow-up was approved by the Belarusian Ministry of Health and received ethical approval from the McGill University Health Centre Research Ethics Board, the Institutional Review Board at Harvard Pilgrim Health Care, and the Avon Longitudinal Study of Parents and Children (ALSPAC) Law and Ethics Committee. A parent or legal guardian provided written informed consent and all participants provided written assent in Russian.

### Assessment of neurocognitive function

Neurocognitive function at the 16-year follow-up was assessed using a computerized battery of the NeuroTrax cognitive tests, previously known as MindStreams tests (NeuroTrax Corp., Modiin, Israel). These tests do not require advanced computer skills and are available in Russian. Moreover, the tests were self-administered, with custom software preinstalled on a polyclinic computer, in the absence of the polyclinic pediatrician or any other test administrator. Pediatricians in PROBIT were not blinded to the intervention arm because they had delivered the postnatal aspect of the intervention. Thus, participant self-administration allowed us to minimize both polyclinic-level clustering due to the pediatrician- and clinic-specific testing environment, as well as potential measurement bias caused by nonblinding of the pediatricians.

The battery consists of 10 short subtests that assess both verbal and nonverbal domains of cognitive function, including immediate and delayed verbal and nonverbal memory, word recognition, executive function, visual–spatial orientation, information-processing speed, and fine motor skills. Age-standardized neurocognitive ability scores were computed from raw data using automatic algorithms and scaled to a conventional IQ-style score, with a mean of 100 and a standard deviation of 15. Standardized scores that measure similar cognitive functions were averaged to produce 7 “index scores” of different domains—memory, executive function, visual–spatial perception, verbal function, attention, information processing, and fine motor skills. A global score was computed by averaging the 7 index scores to reflect general neurocognitive function. Details of the 10 subtests and how the subtests contributed to each of the 7 domain scores are provided in S3 Text. The battery has demonstrated strong reliability and construct validity in identifying different cognitive domains and cognitive deficits in numerous populations, including Russian-speaking populations [15–18].

We preloaded test batteries for all potential participants at each polyclinic onto a laptop computer (HP ProBook 4530S) designated for each pediatrician. We carried out detailed training and follow-up monitoring of the pediatricians to ensure standardized test administration. The study pediatrician arranged for a quiet private testing room, provided brief verbal instructions according to the study protocol, and then left the room to allow each participating child to self-administer the test.

After all visits were completed, we conducted random audit visits to assess the test–retest reliability of the battery, using a different version of the same battery. We randomly selected to return for retesting four participants per pediatrician out of all children seen in follow-up by each of the 24 single-polyclinic pediatricians and three for each of the 14 pediatricians at the 6 high-volume clinics (i.e., 6 total at the polyclinic). A total of 132 participants completed the audit visit within 14 months of the original test on average (interquartile range 11–19 months). The audit visits were carried out by three specially trained Minsk-based physicians who were not involved in primary data collection and were blinded to the measures obtained at the initial visit but not to the experimental or control status of the polyclinic.

### Statistical analysis

Our primary analysis compared mean differences in the global and 7 domain scores between participants in the intervention versus control arms based on intention-to-treat (ITT). We calculated ICCs to assess the within-cluster correlations of each cognitive score. To account for the possible nonindependence of measurements within polyclinics (clusters), we used mixed-effect linear regression to estimate cluster-adjusted mean differences between the two arms according to our study protocol (p. 14, available in S1 Text). We then further adjusted for stratum-level variables (geographic region and urban versus rural location) and individual-level characteristics that are known predictors of cognitive development to minimize imbalances in potential confounders between the two arms due to the relatively small number of clusters (31) in PROBIT (S1 Text, p. 14). Those adjusted variables were age at follow-up, sex, sex- and gestational age-standardized birth weight z-score, maternal age, maternal and paternal education and occupation, parental marital status at birth, maternal smoking during pregnancy, and birth order. We also repeated the ITT analyses for the entire enrolled cohort (*n* = 17,046) after multiple imputation for missing information to examine the robustness of the primary ITT results against potential bias from loss to follow-up. We generated 20 imputed data sets using a chained equation multiple imputation model [19] in SAS (Proc MI) and adjusted the variability in point estimates and standard errors between imputed data according to Rubin’s combination rules using Proc MIANALYZE [20]. This “two-stage” multiple imputation analysis was explicit in our study protocol as a sensitivity analysis to ensure the identical imputed data sets were used for all study outcomes at the 16-year follow-up.

We further analyzed the data using an instrumental variable (IV) approach [21] as a prespecified secondary analysis to account for “non-compliance” with the randomized intervention and estimate the true estimates of breastfeeding effect, rather than the effects of the breastfeeding promotion intervention as estimated in the ITT analysis (study protocol, p. 14–15). IV analysis uses the randomly assigned intervention group as an “instrument” that affects breastfeeding but has no independent effect on neurocognitive function. As with the ITT analysis, we first assessed cluster-adjusted IV estimates, then adjusted for the same stratum- and individual-level characteristics. We also performed a standard observational (as-fed) analysis using multivariable linear regression models, controlling for the same baseline characteristics, to examine associations of socioeconomic and other family characteristics with the cognitive scores and compare their magnitudes with those of breastfeeding effects. Both the IV and as-fed analyses were based on exclusive breastfeeding ≥3 versus <3 months because the intervention was to promote both exclusivity and duration of breastfeeding, and we observed the largest contrast in exclusive breastfeeding ≥3 versus <3 months between the two randomized groups. However, we also performed sensitivity analyses using different cutoffs of exclusive breastfeeding duration.

Finally, we conducted additional sensitivity analyses to assess robustness of the primary analysis results. First, we repeated our analyses after excluding those children who did not complete the neurocognitive test on a single day (*n* = 99). Second, we estimated the associations stratified by the participants’ knowledge about their trial arm to examine whether their knowledge biased the cognitive performance. All analyses except multiple imputations were performed using Stata/SE version 14 (Stata Corp).

## Results

A total of 13,557 participants were examined at a median age of 16.1 years (SD 0.53, IQR 15.8–16.4) at the follow-up (Fig 1). The 16-year follow-up rates were similar in the intervention (79.7%) and control (79.3%) groups. Of those followed up, 130 (0.9% overall; 1.4% and 0.5% in the intervention and control group, respectively) children were unable or unwilling to take the computerized neurocognitive test because of diagnosed neuropsychiatric disorder (*N* = 26), serious vision problems (*N* = 1), or other nonspecified reasons (*N* = 103). This exclusion resulted in a total of 13,427 (99% of those followed up) children as the analytic sample for the present study.

Overall, baseline and follow-up characteristics of children with the cognitive scores were similar between the intervention and control groups (Table 1); minor differences were consistent with those reported at recruitment [13]. ICCs (as test–retest reliability measures) between the NeuroTrax testing scores for the study visit and the audit visit among the 132 children in the audit sample ranged from ICC = 0.4 for the memory and attention scores to ICC = 0.7 for the global score (S1 Table). The estimated ICCs and confidence intervals were similar between the intervention and control groups. NeuroTrax testing scores were modestly correlated (correlation coefficients range between 0.14–0.31) with the pediatrician-administered WASI scores previously measured at age 6.5 years (S1 Table).

**Table 1:** Baseline and follow-up characteristics of 13,427 participants with the cognitive scores at 16-year follow-up by intervention group (*N*, percentage).

Characteristics	Intervention	Control
	(*N* = 6,967)	(*N* = 6,460)
Male sex	3,539 (50.8)	3,361 (52.0)
Gestational age, weeks		
37–38	1,147 (16.4)	1,357 (21.0)
39–41	5,719 (82.1)	5,026 (77.8)
42–43	101 (1.5)	79 (1.2)
Birthweight, mean (SD), g	3,443 (417)	3,441 (421)
Age at PIV follow-up, years, mean (SD)	16.2 (0.5)	16.1 (0.4)
Maternal age, years		
<20	970 (13.9)	840 (13.0)
20–34	5,711 (82.0)	5,354 (82.9)
≥35	286 (4.1)	266 (4.1)
Maternal education		
Completed university	978 (14.0)	835 (12.9)
Partial university	3,324 (47.7)	3,545 (54.9)
Secondary	2,375 (34.1)	1,901 (29.4)
Incomplete secondary	290 (4.2)	179 (2.8)
Paternal education*		
Completed university	916 (13.6)	798 (12.8)
Partial university	2,870 (42.5)	3,283 (52.7)
Secondary	2,799 (41.4)	2,040 (32.7)
Incomplete secondary	172 (2.5)	112 (1.8)
Maternal occupation at birth		
Nonmanual	2,875 (41.3)	2,993 (46.3)
Manual	2,419 (34.7)	2,089 (32.3)
Unemployed	1,673 (24.0)	1,378 (21.3)
Paternal occupation at birth		
Nonmanual	1,767 (25.4)	2,037 (31.5)
Manual	3,919 (56.2)	3,280 (50.8)
Unemployed	911 (13.1)	914 (14.1)
Unknown	370 (5.3)	229 (3.5)
Maternal marital status at birth		
Married	6,106 (87.6)	5,910 (91.5)
Cohabitating	567 (8.1)	334 (5.2)
Unmarried	294 (4.2)	216 (3.3)
Older siblings		
0	4,094 (58.8)	3,540 (54.8)
1	2,331 (33.4)	2,338 (36.2)
≥2	542 (7.8)	582 (9.0)
Younger siblings, measured at age 6.5 years**		
0	4,707 (76.9)	4,614 (74.3)
1	1,289 (21.1)	1,416 (22.6)
≥2	121 (2.0)	187 (3.0)
Maternal smoking during pregnancy, yes	160 (2.3)	104 (1.6)
Duration of exclusive breastfeeding, months***		
<3	3,777 (54.8)	6,009 (93.1)
≥3	3,111 (45.2)	444 (6.9)
Duration of any breastfeeding, months****		
<3	2,056 (29.8)	2,595 (40.2)
3–<6	1,566 (22.7)	1,511 (23.4)
**> =** 6	3,271 (47.5)	2,344 (36.3)
Location of residence		
East, urban	2,168 (31.1)	1,926 (29.8)
East, rural	1,065 (15.3)	1,073 (16.6)
West, urban	2,262 (32.5)	1,222 (18.9)
West, rural	1,472 (21.1)	2,239 (34.7)

* Missing for 437 participants

** Missing for 1,103 participants

*** Missing for 86 participants

**** Missing for 84 participants

Table 2 shows the means of neurocognitive function scores in the intervention and control group and estimated differences between the two groups from the ITT analysis without multiple imputation, along with the ICCs. Within-polyclinic clustering of neurocognitive function scores was low (ICCs ranged from 1% to 3% of the total variance in the scores being at polyclinic level), suggesting that the different pediatricians’ and polyclinics’ characteristics did not affect the test scores. Overall, neurocognitive scores of children in the intervention group were almost identical to those of the control group, except for slightly higher scores in verbal function and memory. The cluster-adjusted mean differences between the two groups were 1.5 (95% CI −0.04–3.0) points higher for verbal function and 1.2 (95% CI −0.1–2.4) points higher for memory score. After adjusting for baseline characteristics, the observed mean differences were 1.4 (95% CI 0.3–2.5) for verbal function and 1.2 (95% CI 0.01–2.4) for memory. Results remained substantially unchanged in the sensitivity analysis after excluding the approximately 100 children who did not complete the test or completed it after an interruption for any reason: The cluster- and baseline characteristics-adjusted mean differences between the two groups were 0.8 (95% CI −0.7–2.3) for global score, 1.2 (95% CI −0.01–2.4) for memory, and 1.4 (95% CI 0.4–2.5) for verbal function. In addition, the ITT analysis stratified by the participants’ knowledge of their randomized group assignment showed that the observed differences were unlikely to be biased by nonblinding of the participants. Among children who did not identify their randomly assigned group correctly (*N* = 9495, 71.1%), the mean differences in verbal function between the intervention and control groups were 1.6 (95% CI −0.1–3.3) in the cluster-adjusted estimation and 1.4 (95% CI 0.3–2.5) in the further-adjusted model. Of the 3,858 children who correctly identified their group assignment, the corresponding figures were 2.4 (95% CI 0.5–4.2) and 1.9 (95% CI 0.4–3.4). The interaction *p*-values for group assignment and participant knowledge were 0.92 for the cluster-adjusted and 0.47 for the further-adjusted models. The ITT analyses based on the multiple imputed data (S2 Table) also showed consistent results—no overall differences but a slightly higher verbal function score favouring the intervention group (3.0 points [95% CI −0.01–6.0] in cluster-adjusted analysis; 2.5 points [95% CI 0.2–4.8] after further adjustment for baseline characteristics).

**Table 2:** Intraclass correlation and ITT analysis of mean differences (95% CI) of neurocognitive scores at age 16 years in treatment (*N* = 6,967) versus control (*N* = 6,460) groups.

Cognitive domain	ICC	Mean (SD) in the intervention group	Mean (SD) in the control group	Cluster-adjusted mean difference	Further-adjusted (for baseline characteristics*) mean difference
Global score	0.03	100.4 (14.6)	99.6 (15.4)	1.0 (−1.0–3.1)	0.8 (−0.6–2.3)
Memory	0.01	100.5 (14.8)	99.5 (15.2)	1.2 (−0.1–2.4)	1.2 (0.01–2.4)
Executive functioning	0.03	100.2 (15.0)	99.8 (15.0)	0.3 (−1.7–2.3)	−0.03 (−1.3–1.2)
Visual spatial	0.02	99.9 (15.0)	100.1 (15.0)	0.1 (−1.6–1.9)	−0.1 (−1.3–1.2)
Verbal function	0.02	100.7 (14.7)	99.3 (15.2)	1.5 (−0.04–3.0)	1.4 (0.3–2.5)
Attention	0.02	100.3 (14.7)	99.6 (15.3)	0.7 (−1.1–2.6)	0.4 (−0.8–1.7)
Information processing speed	0.01	100.4 (14.9)	99.6 (15.1)	0.7 (−0.7–2.2)	0.5 (−0.5–1.5)
Motor skills	0.03	99.7 (15.2)	100.3 (14.7)	−0.6 (−2.6–1.3)	−0.5 (−2.4–1.3)

* Baseline factors adjusted for include stratum-level variables, age at neurocognitive test, sex, maternal age, maternal and paternal education and occupation, maternal marital status, birthweight, and number of older siblings.

**Abbreviations:** ITT, intention-to-treat SD, standard deviation.

Table 3 presents the IV estimation of effects of exclusive breastfeeding ≥3 months (versus <3 months) on neurocognitive function. After adjusting for clustering and potential confounding factors, children who were breastfed exclusively for 3 or more months had 3.5-point (95% CI 0.9–6.1) higher verbal function and 3.1-point (95% CI −0.5–6.7) higher memory scores than those who were breastfed exclusively for less than 3 months. Global and other domain scores showed little statistical evidence to support observed differences between the two groups. Consistent results—slightly higher verbal function scores in children breastfed but no statistical support for differences in other domain and global scores—were observed when we analyzed the number of months of exclusive breastfeeding (3.0 [95% CI 0.8–5.2] points higher verbal function) or any breastfeeding for ≥6 months (9.4 [95% CI 2.8–16.0] points higher verbal function than breastfeeding <6 months) as breastfeeding exposure in IV analysis.

**Table 3:** IV estimates of breastfeeding effects (95% CI) on neurocognitive scores at age 16 years by breastfeeding exclusivity and duration, (*N* = 12,912).

Cognitive domain	Cluster-adjusted mean difference (exclusive breastfeeding ≥3 versus <3 months)	Further-adjusted (for baseline characteristics) mean difference (exclusive breastfeeding ≥3 versus <3 months)
Global score	2.7 (−3.0–8.4)	2.2 (−3.6–8.1)
Memory	3.1 (−0.7–6.9)	3.1 (−0.5–6.7)
Executive functioning	0.7 (−4.5–5.9)	−0.03 (−4.4–4.3)
Visual spatial	0.3 (−4.3–4.9)	−0.1 (−5.4–5.2)
Verbal function	3.9 (−0.5–8.3)	3.5 (0.9–6.1)
Attention	1.9 (−3.2–7.1)	1.2 (−4.7–7.1)
Information processing	2.0 (−2.1–6.1)	1.3 (−4.3–7.0)
Motor skills	−1.7 (−6.7–3.2)	−1.4 (−8.2–5.4)

**Abbreviation:** IV, instrumental variable.

Observational analyses based on maternal reports of exclusive breastfeeding showed that, after adjusting for cluster and potential confounders, exclusive breastfeeding for 3 months or longer (versus <3 months) yielded little beneficial effect on neurocognitive function, including verbal function at age 16 years (Table 4 and S3 Table for domain-specific scores). In contrast, family and birth characteristics other than breastfeeding had larger effect sizes and statistical evidence to support their associations with neurocognitive test scores at age 16 years. In particular, children whose parents had less than a secondary education had global cognitive scores that were 5–6 points (95% CIs 3.6–7.1 and 4.2–8.1 for maternal and paternal education, respectively) lower than those whose parents had a university degree. Moreover, the associations of maternal and paternal education were independent and consistent in patterns with each other (Table 4). Birth order showed negative, graded associations with cognitive scores: Compared to first-born children, second-born children had a 1.5-point (95% CI 0.9–2.0) lower global score, and third- or later-born children had a 5.1-point (95%CI 4.1–6.1) lower global score. These patterns were consistent across all domain scores.

**Table 4:** Observational analysis of multivariable associations of exclusive breastfeeding (≥3 versus <3 months) and nonbreastfeeding factors (mean differences and 95% CI) with global cognitive score at age 16 years (without multiple imputation).

	Adjusted mean difference (95% CI): Global neurocognitive score
Exclusive breastfeeding	
< 3 months	reference
≥3 months	0.2 (−0.4–0.9)
Age at test, years	1.1 (0.6–1.6)
Male	−0.8 (−1.3–−0.3)
Birth weight z-score	0.7 (0.5–1.0)
Maternal age, years	
<20	−0.7 (−1.5–0.1)
20–34	reference
≥35	0.6 (−0.7–1.9)
Maternal education	
Completed university	reference
Partial university	−2.5 (−3.3–−1.6)
Secondary	−4.4 (−5.4–−3.4)
Incomplete secondary	−5.3 (−7.1–−3.6)
Paternal education	
Completed university	reference
Partial university	−1.5 (−2.4–−0.6)
Secondary	−2.3 (−3.3–−1.3)
Incomplete secondary	−6.1 (−8.1–−4.2)
Maternal occupation at birth
Nonmanual	reference
Manual	−2.5 (−3.2–−1.8)
Unemployed	−1.1 (−1.9–−0.4)
Paternal occupation at birth	
Nonmanual	reference
Manual	−2.0 (−2.7–−1.4)
Unemployed	−1.2 (−2.0–−0.3)
Unknown	−0.5 (−2.8–1.7)
Maternal marital status at birth	
Married	reference
Cohabitating	−1.6 (−2.6–−0.6)
Unmarried	−0.5 (−3.0–1.9)
Birth order	
1st	reference
2nd	−1.5 (−2.0–−0.9)
≥3rd	−5.1 (−6.1–−4.1)
Location of residence	
East, urban	reference
East, rural	−2.0 (−4.1–0.1)
West, urban	−0.7 (−2.9–1.5)
West, rural	−2.9 (−4.8–−0.9)

## Discussion

In this follow-up of PROBIT participants at age 16 years, children in the intervention group showed no difference in overall neurocognitive function but slightly higher scores for verbal function. Our conclusion that the randomized intervention had a beneficial effect on verbal function, but not on other domains, is based not only on statistical significance but also on the patterns of results from our prespecified ITT and IV analyses and sensitivity analyses to explore the potential impacts of biases [22–24]. Verbal function was the cognitive domain that showed beneficial effects in both our prespecified primary ITT and secondary IV analyses. Although the ITT analysis reached the conventional statistical significance only after adjusting for baseline characteristics, importantly, the point estimates remained unchanged between the adjusted and nonadjusted models. Thus, adjustment of known predictors of cognitive function improved the statistical precision without changing the effect sizes. ITT analysis estimated the effects of breastfeeding promotion intervention rather than the effects of breastfeeding per se, and thus, it may underestimate effects of breastfeeding itself due to “noncompliance” with the randomized intervention. Our IV estimation of the effect of exclusive breastfeeding for ≥3 months to account for this noncompliance (i.e., overlap in breastfeeding between the randomized groups) further supports the conclusion. Moreover, higher verbal function scores were also found when different cut-offs of breastfeeding were used in our IV sensitivity analyses. This finding is consistent with the results we observed at age 6.5 years, when the strongest benefit was for verbal IQ [11]. Although the observed mean differences at age 16 in ITT analysis were relatively small in magnitude, the point estimates lie within the 95% CI around the estimated differences observed at 6.5 years of age. It is also important to note that the statistically significant effect was consistently observed in verbal function among multiple neurocognitive domains across different statistical models, despite the correlations between the domain scores (correlation coefficients ranged from 0.15 to 0.71).

Results of the present analysis, combined with those of the 6.5-year follow-up, suggest that the beneficial effects of breastfeeding on verbal ability persist at older ages, however, with a substantially reduced magnitude. The magnitude of the observed effects of breastfeeding is also relatively modest compared with those of other environmental factors at age 16, such as family socioeconomic position. At age 6.5 years, children in the intervention group showed a 7.9-point (95% CI 1.3–14.2) higher verbal IQ (ITT estimation), a similar difference as that observed with maternal education: 8.4 (95% CI 6.9–10.1) points higher in children of mothers with university education (versus less than secondary education). The corresponding differences at age 16 years were 1.4 (95% CI 0.3–2.5) points higher verbal function for the breastfeeding promotion intervention and 3.8 (95% CI 2.0–5.6) points for the same contrast in maternal education. It is of note that the effects of breastfeeding were not modified by maternal education (all *p*-values for interactions >0.4). A decreasing magnitude of breastfeeding effects with advancing child age is consistent with the results of a recent meta-analysis [2] showing a smaller effect of breastfeeding on IQ among adolescents (1.9-point increase in IQ) than among younger children (4.1-point increase). Given the observed decrease in cognitive benefits over time and the modest effect sizes in adolescence, those benefits should not be interpreted as substantial impacts at the individual level. Further research examining other outcomes related to neurocognitive function, including educational attainment and lifestyle behaviors [25], would shed additional light on the long-term neurocognitive benefits of breastfeeding.

The mechanisms underlying a “diluted” effect of breastfeeding at later ages are unclear, but we can speculate about possible reasons. Twin and adoption studies have reported that genetic effects on neurocognitive function increase with age, with an estimated heritability of 0.4 during childhood versus 0.8 at maturity [26,27]. Alternatively, other environmental factors such as school characteristics, peer influences, and parental intellectual stimulation may become more important as children age. Although direct comparison of genetic versus environmental effects is not feasible in our data, our multivariable regression analysis of nonbreastfeeding factors supports the importance of sociodemographic exposures for neurocognitive development.

Studies of infant feeding (breastmilk versus preterm formula and preterm versus standard formula) have reported that early diet is more strongly associated with language development [28]. Nutrients in breastmilk such as docosahexaenoic acid (DHA) and arachidonic acid (AA) may be beneficial for cognitive development as shown in some [29,30] (but not all [31]) studies, although evidence of mechanisms specific to verbal function is absent. Better verbal function in breastfed children may also be explained by greater maternal responsiveness to infants, greater psychological bonding of the mother–infant dyad, or more verbal exchange during breastfeeding compared with bottle feeding [32,33].

Our study is the largest randomized trial in the area of human lactation, with a high follow-up rate to age 16 years. Use of a computerized, self-administered neurocognitive test resulted in high statistical power, owing to low within-polyclinic ICCs (0.01–0.03). More importantly, the computerized test minimized any potential influence of the study pediatricians who were not blinded to the intervention status of their children. Breastfeeding effects estimated with multiple analytical approaches, including the traditional ITT analysis based on the complete cases and multiply imputed data, standard observational data analysis according to maternal report of breastfeeding, and IV estimation provide an improved understanding of the causal role of breastfeeding in child neurocognitive development. In addition, our results showed no evidence of the potential bias due to unblinding of the study children with the intervention assignment.

Limitations of our study should also be considered in interpreting our results. The test–retest reproducibility of the neurocognitive test estimated in the audit sample was modest (ranges 0.4–0.7) compared to that of the pediatrician-administered test at age 6.5 years (ranges 0.6–0.7). This modest reproducibility is presumably partly due to error in measuring cognitive ability, and this would have reduced the precision of our estimates. But it may also reflect a trade-off. The computer-assisted, self-administered test at age 16 sharply reduced the high within-polyclinic clustering and the possible bias due to nonblinding of the pediatricians. On the other hand, it may have resulted in participants’ inconsistent efforts due to self-administration of the test without supervision and consequently increased measurement errors. The measurement errors may have also contributed to the “diluted” effects of breastfeeding owing to nondifferential misclassification. Correlations between WASI and NeuroTrax test scores were also low to modest (ranges 0.14–0.31). Differences in the mode of testing (paper and pencil versus computerized and pediatrician- versus self-administered), different testing batteries, and the 10-year age gap between the two visits all may have contributed to the modest correlations. Nevertheless, our observed correlations between WASI and NeuroTrax scores are comparable to other studies employing different test batteries over time [34]. Moreover, the NeuroTrax test scores were strongly associated with parental socioeconomic factors, birth order, and birth weight, and all these observed associations were in the expected direction. Finally, it should be noted that our study participants were restricted to healthy infants with “normal” birth weight born at term. Thus, the effects of the breastfeeding promotion intervention observed in our study might be different from those among infants born preterm or with low birth weight.

In conclusion, our randomized intervention to promote prolonged and exclusive breastfeeding showed little evidence on beneficial effect of breastfeeding on overall neurocognitive function at age 16 years. However, we observed slightly higher verbal function at age 16 years, suggesting limited but persistent benefit to verbal ability. Nevertheless, these benefits were small in magnitude compared to other family and birth factors and appeared to decrease with age from childhood to adolescence.

## Supporting information

S1 Table ICCs (95% CI) of the NeuroTrax testing scores with the 16-year audit scores and correlation coefficients (95% CI) with WASI scores at age 6.5 years. ICC, intraclass correlation coefficient; WASI, Wechsler Abbreviated Scale of Intelligence.
(DOCX)

S2 Table ITT analysis of mean differences (95% CI) of neurocognitive scores at age 16 years in treatment (*N* = 8,865) versus control (*N* = 8,181) groups with multiple imputation. ITT, intention-to-treat.
(DOCX)

S3 Table Observational analysis of associations between exclusive breastfeeding ≥3 versus <3 months and neurocognitive scores at age 16 years (without multiple imputation) (*N* = 12,912). (DOCX)

S1 Text PROBIT 16-year follow-up study protocol. PROBIT, promotion of breastfeeding intervention trial.
(DOCX)

S2 Text CONSORT statement. (DOC)

S3 Text Description of 10 subtests and scoring of 7 index scores of specific cognitive domains in NeuroTrax tests. (DOC)

## Abbreviations

AA: arachidonic acid
ALSPAC: Avon Longitudinal Study of Parents and Children
DHA: docosahexaenoic acid
ICC: intraclass correlation coefficient
IQ: intelligence quotient
ITT: intention-to-treat
IV: instrumental variable
PROBIT: Promotion of Breastfeeding Intervention Trial
WASI: Wechsler Abbreviated Scale of Intelligence
