# Supplementary material for: Breastfeeding during infancy and neurocognitive function in adolescence: 16-year follow-up of the PROBIT cluster-randomized trial
Source: PLoS Med. 2018 Apr 20;15(4):e1002554. doi: 10.1371/journal.pmed.1002554 (PMC5909901; doi:10.1371/journal.pmed.1002554)
Supplement: S1 Table — ICC, intraclass correlation coefficient; WASI, Wechsler Abbreviated Scale of Intelligence. (DOCX) [file pmed.1002554.s001.docx]

S1 Table Intraclass correlation coefficients (95% CI) of the NeuroTrax testing scores with the 16-year audit scores and correlation coefficients (95% CI) with Wechsler Abbreviated Scale of Intelligence (WASI) scores at age 6.5 years

|  | NeuroTrax same domain score  at the audit | WASI  Full-scale IQ | WASI  Verbal IQ | WASI  Performance IQ |
| --- | --- | --- | --- | --- |
|  | N=132 | N=12372 | N=12376 | N=12383 |
| Global score | 0.72 (0.63, 0.80) | 0.31 (0.29, 0.33) | 0.26 (0.25, 0.28) | 0.29 (0.27, 0.30) |
| Memory | 0.39 (0.25, 0.54) | 0.21 (0.20, 0.23) | 0.17 (0.16, 0.19) | 0.21 (0.19, 0.23) |
| Executive functioning | 0.46 (0.32, 0.59) | 0.24 (0.22, 0.25) | 0.21 (0.19, 0.22) | 0.21 (0.20, 0.23) |
| Visual spatial | 0.55 (0.43, 0.67) | 0.20 (0.18, 0.22) | 0.16 (0.14, 0.17) | 0.21 (0.19, 0.22) |
| Verbal function | 0.48 (0.35, 0.61) | 0.24 (0.22, 0.25) | 0.22 (0.20, 0.23) | 0.20 (0.18, 0.22) |
| Attention | 0.39 (0.24, 0.53) | 0.21 (0.19, 0.22) | 0.18 (0.16, 0.19) | 0.19 (0.17, 0.20) |
| Information processing | 0.54 (0.41, 0.66) | 0.21 (0.19, 0.23) | 0.16 (0.14, 0.18) | 0.21 (0.19, 0.22) |
| Motor skills | 0.49 (0.36, 0.62) | 0.16 (0.15, 0.18) | 0.14 (0.13, 0.16) | 0.15 (0.13, 0.17) |
