# Supplementary material for: Breastfeeding during infancy and neurocognitive function in adolescence: 16-year follow-up of the PROBIT cluster-randomized trial
Source: PLoS Med. 2018 Apr 20;15(4):e1002554. doi: 10.1371/journal.pmed.1002554 (PMC5909901; doi:10.1371/journal.pmed.1002554)
Supplement: S2 Table — ITT, intention-to-treat. (DOCX) [file pmed.1002554.s002.docx]

S2 Table. Intention-to-treat analysis of mean differences (95% CI) of neurocognitive scores at age 16 years in treatment (N=8865) vs. control (N=8181) groups with multiple imputation

| Cognitive domain | Cluster-adjusted  mean difference | Further adjusted (for baseline characteristics*)  mean difference |
| --- | --- | --- |
| Global score | 1.1 (-0.7, 2.9) | 0.6 (-0.6, 1.9) |
| Memory | 1.5 (-0.1, 3.1) | 1.3 (-0.1, 2.9) |
| Executive functioning | 0.7 (-1.5, 2.8) | 0.1 (-1.3, 1.5) |
| Visual spatial | 0.7 (-2.3, 3.7) | -0.1 (-2.2, 2.1) |
| Verbal function | 3.0 (-0.01, 6.0) | 2.5 (0.2, 4.8) |
| Attention | 0.8 (-1.0, 2.7) | 0.3 (-0.9, 1.6) |
| Information processing speed | 0.8 (-0.5, 2.0) | 0.4 (-0.5, 1.4) |
| Motor skills | -0.1 (-2.1, 1.9) | -0.4 (-2.2, 1.4) |

* Baseline factors adjusted for include stratum-level variables, age at neurocognitive test, sex, maternal age, maternal and paternal education and occupation, maternal marital status, birthweight, and number of older siblings
