# Supplementary material for: Breastfeeding during infancy and neurocognitive function in adolescence: 16-year follow-up of the PROBIT cluster-randomized trial
Source: PLoS Med. 2018 Apr 20;15(4):e1002554. doi: 10.1371/journal.pmed.1002554 (PMC5909901; doi:10.1371/journal.pmed.1002554)
Supplement: S3 Table — (DOCX) [file pmed.1002554.s003.docx]

S3 Table. Observational analysis of associations between exclusive breastfeeding ≥3 vs. <3 months and neurocognitive scores at age 16 years (without multiple imputation), (N=12,912)

| Cognitive domain | Cluster-adjusted  mean difference  (exclusive breastfeeding ≥3  vs. < 3 months) | Further adjusted (for baseline characteristics)  mean difference  (exclusive breastfeeding ≥3  vs. < 3 months) |
| --- | --- | --- |
| Global score | 0.8 (0.1, 1.4) | 0.2 (-0.4, 0.9) |
| Memory | 0.5 (-0.2, 1.1) | 0.1 (-0.5, 0.8) |
| Executive functioning | 0.4 (-0.2, 1.1) | -0.03 (-0.7, 0.6) |
| Visual spatial | 0.4 (-0.2, 1.1) | 0.1 (-0.5, 0.8) |
| Verbal function | 0.8 (0.2, 1.5) | 0.5 (-0.2, 1.1) |
| Attention | 0.4 (-0.2, 1.1) | 0.1 (-0.5, 0.8) |
| Information processing | 0.2 (-0.5, 0.9) | -0.1 (-0.7, 0.6) |
| Motor skills | 0.4 (-0.2, 1.1) | 0.1 (-0.5, 0.76) |
