# Supplementary material for: Breastfeeding during infancy and neurocognitive function in adolescence: 16-year follow-up of the PROBIT cluster-randomized trial
Source: PLoS Med. 2018 Apr 20;15(4):e1002554. doi: 10.1371/journal.pmed.1002554 (PMC5909901; doi:10.1371/journal.pmed.1002554)
Supplement: S1 Text — PROBIT, promotion of breastfeeding intervention trial. (DOCX) [file pmed.1002554.s004.docx]

**PROBIT IV**

**Breastfeeding Promotion RCT:**

**PROTOCOL OF RESEARCH PROJECT**

**1. Specific Aims**

Breastfeeding improves health outcomes in infancy, including risks for atopic eczema (syn. ‘atopic dermatitis’, ‘eczema’) and gastrointestinal infections, but the long-term physiological effects of breastfeeding on child health are less clear. Among the most controversial purported benefits of breastfeeding are long-term effects on adiposity, blood pressure, neurocognitive function and asthma. Existing evidence is largely based on observational studies, which are prone to measurement error, publication bias and reverse causality. In addition, substantial differences between mothers who do and do not choose to breastfeed may confound results from observational studies, so it is difficult to determine whether the observational associations of breastfeeding duration with child health outcomes are causal or have alternative explanations.

The unbiased effects of breastfeeding can probably only be convincingly demonstrated in a randomized controlled trial (RCT). While it is not feasible to randomize healthy term infants to be breast or bottle fed, it is possible to randomize mother-child pairs to a breastfeeding promotion intervention. The Promotion of Breastfeeding Intervention Trial (PROBIT, ISRCTN37687716), the largest randomized trial of breastfeeding ever conducted, successfully increased breastfeeding duration and exclusivity among over 17,000 mother-infant pairs. The PROBIT intervention resulted in high rates of prolonged and exclusive breastfeeding; for example at 3 months, 43% of intervention mothers were exclusively breastfeeding compared with 6% of mothers in the control arm. The most plausible mechanism for any observed differences between the intervention groups is that the increased breastfeeding, caused by the breastfeeding promotion intervention, is the cause of differences between the intervention groups. Previous phases of PROBIT have demonstrated high rates of follow-up and valid, research-standard measurement of outcomes. The PROBIT trial thus offers a unique opportunity to obtain un-confounded estimates of the influence of breastfeeding on child health outcomes.

Our current proposal is based on the 15-16 year follow-up of children enrolled in PROBIT. Our hypotheses build on important findings from a previous follow-up of PROBIT children. In an intention-to-treat analysis, 6.5-year olds in the intervention arm had a 7.5 point advantage in verbal intelligence quotient (IQ) and higher teacher rated reading and writing *vs.* controls. At that age, most children had recently started school, but IQ is sensitive to environmental factors such as length of schooling. Thus, it remains unclear whether breastfeeding has an enduring effect on IQ. Furthermore, the pediatricians administering the IQ tests were not blinded to the children’s randomization status. In our current proposal, we plan to use self-administered tests of cognition and language, which will minimize any administrator bias. Also at age 6.5 years, the PROBIT intervention was not associated with asthma assessed by questionnaire (odds ratio: 1.2; 95% CI: 0.7-1.9), but the wide confidence interval does not exclude potentially important protective or adverse effects. In the current proposal, we include spirometric lung function measures, which index susceptibility to, and consequences of, asthma. Spirometry provides objective and continuous outcome measures, increasing study power. The PROBIT intervention was associated with a reduced risk of atopic eczema at 12 months (odds ratio: 0.54, 95% CI, 0.31-0.95), but was not associated with eczema assessed by questionnaire at 6.5 years (odds ratio: 1.0, 95% CI, 0.5-1.8). However, the reported prevalence of eczema at age 6.5 was also extremely low (1%). We hypothesized that parents may not have reported cases of eczema that were not diagnosed by a doctor. In this proposal, we plan to minimize such underreporting by training the study pediatricians to conduct physical examinations of participant’s skin to identify atopic eczema around the participant’s eyes, neck, elbows, knees or ankles. Such examinations should provide a more systematic approach to identifying true cases of atopic eczema.

Also at age 11.5 years, the PROBIT intervention was not associated with measured obesity (odds ratio: 1.17 (95% CI, 0.97 to 1.41) or blood pressure (mean difference between intervention vs control arms:

1.0 mmHg (95% CI: -1.1 to 3.1) for systolic and 0.8 mmHg (-0.6 to 2.3) for diastolic blood pressure, but the wide confidence interval does not exclude potentially important protective or adverse effects.

The **specific aims** of the current proposal are to perform an intention-to-treat analysis comparing the randomized intervention *vs.* control arm. We will estimate the unconfounded, causal effects of breastfeeding promotion for the following hypotheses:

1. Prolonged, exclusive breastfeeding improves neurocognitive outcomes, including verbal and non-verbal cognitive ability and vision at age 15-16 years.
2. Prolonged, exclusive breastfeeding improves spirometric lung function and reduces rates of atopic eczema at age 15-16 years.
3. Prolonged, exclusive breastfeeding reduces adiposity and blood pressure at age 15-16 years.

We will also use data already collected as part of the PROBIT study to perform observational analyses examining associations of early life exposures, including birth characteristics and infant growth and feeding, with child health outcomes at age 6.5, 11 and 15-16 years, including cognition, asthma, atopy, dental health, adiposity, blood pressure, bloodspot measures of cardiometabolic health, neurocognitive outcomes, and lung function.

Furthermore, we will analyze associations of treatment assignment and breastfeeding duration with maternal health and adiposity at 11 years postpartum, using data already collected as part of PROBIT III.

The proposed research will provide an estimate of the causal effect of breastfeeding on outcomes meaningful for health in childhood and throughout the lifecourse. Understanding the causal effects of breastfeeding is important to maximize the population-wide impact of guidelines focusing on early nutrition, by providing robust data, ensuring consistent conclusions, and minimizing potential harms. Although different in many socioeconomic, cultural, and economic respects from the United States, Belarus is a relatively developed country, with strict hygienic standards, high immunization rates, low incidence of infection, low rates of infant and child mortality, similar types of formula feeds and accessible health care services. The results of this current proposal are therefore likely to inform policy in the US and many other countries.

**2. Research Strategy**

**2.1. Significance**

Breastfeeding is widely promoted as a cornerstone of public health.^1^ Observational studies, including several involving co-applicants on the current proposal, suggest that being breastfed reduces risks for obesity, hypertension, diabetes, heart disease, asthma and atopy. ^2-8^ Yet mothers who breastfeed differ from mothers who formula feed: they are wealthier, better educated, less likely to smoke, and more likely to engage in other beneficial health behaviors.^9,10^ Thus, residual or unmeasured confounding, rather than breastfeeding itself, may explain observed associations between breastfeeding and health outcomes. To disentangle the health effects of breastfeeding from the characteristics of mothers who choose to breastfeed, we will perform an intention-to-treat analysis of a breastfeeding promotion intervention on neurocognitive and lung function outcomes in a large, well-characterized population of children followed from birth. Each of the outcomes to be studied are known to be sensitive to early life exposures, which can permanently program organ structure and function resulting in profound influences on health, function, and mortality risks throughout the lifecourse. In the sections below, we summarize the current evidence for associations of breastfeeding with each of the primary outcomes of this proposal, and highlight areas needing further study.

**2.1.1. Neurocognitive function**

Brain development is fastest in the 3^rd^ trimester of gestation and the first 4 years after birth^11^ and is influenced by nutrition.^12-15^ Observational studies report consistently higher intelligence quotient (IQ) scores of 2 to 5 points in breast- *vs* formula-fed term infants.^16^ However, although many observational studies control for socioeconomic status and maternal intelligence, uncontrolled confounding by subtle differences in the mother’s behavior or her interaction with the infant are possible. In the 6.5 year follow-up of PROBIT, IQ was measured by the Wechsler Abbreviated Scales of Intelligence (WASI).^17^ Children randomized to the breastfeeding promotion arm had a 7.5 (95% CI: 0.8 to 14.3) point advantage in verbal IQ, 2.9 (-3.3 to 9.1) point difference for performance IQ and 5.9 (-1.0 to 12.8) point difference for full-scale IQ, *vs* controls.^17^ Smaller, but consistently positive, differences of 2 to 3 IQ points were seen in a blinded audit of 190 children and blinded teacher rated academic performance. Our experimental data, based on strict randomization and intention-to-treat analysis, is therefore consistent with observational data^16^ and suggests that associations in term infants are causal.^17,18^

Nevertheless, these findings have been controversial and merit further investigation. Our current proposal maintains the strengths of earlier work within PROBIT, i.e. the large sample size and randomized design, while improving on the prior limitations, namely the young age at outcome assessment and imprecisely measured outcomes. An observational study and meta-analysis^19^ found that a positive association of breastfeeding with child IQ became negligible after controlling for parental IQ. Other studies that controlled for maternal IQ, however, report persistent, albeit attenuated, IQ benefits.^16,20^ In an RCT like PROBIT, parental IQ should be distributed randomly between treatment groups, as demonstrated for other measured baseline characteristics,^21^ and thus should not confound the intervention effect. The effect sizes for IQ at age 6.5 in PROBIT were imprecisely measured, due to high within-pediatrician clustering of IQ values across the 31 trial sites.^22^ While analyses based on the blinded audit and teacher ratings suggest the direction of the observed differences is robust, we do not know whether the large difference in verbal IQ arose by chance or a systematic overestimate of verbal IQ in the experimental group. The smaller differences seen in the blinded audit and teacher ratings may more closely estimate the true effect. All pediatricians were trained, monitored, and audited in their administration of the WASI, and no beneficial intervention effects were observed for other outcomes,^23-25^ equally subject to systematic error, providing some reassurance that the pediatricians’ assessments of outcomes were unbiased. Nevertheless, the pediatricians who administered the WASI were not blinded to the trial arm of the children they examined, as many had contributed to the original intervention by providing postnatal support and encouragement of prolonged, exclusive breastfeeding.^21^ It is possible that the pediatricians at the intervention sites may have differentially administered or scored the WASI. In addition, about 20% of children seen at age 6.5 years had yet to start school, and they may have had difficulty completing the IQ test, potentially generating random measurement error.

Our current proposal builds on earlier findings by determining, in an intention-to-treat analysis that preserves the original random assignment, the magnitude of effect of the intervention on child cognition at age 15-16 years. We will use a computerized cognitive assessment battery that each child will self-complete with minimal instruction and automated scoring. This approach will counter the potential subjectivity in scoring WASI assessments, which could have compounded both the lack of blinding among pediatricians (leading to systematic error) and random error (thus reducing power^22^). The children will be older and better able to participate in testing. Few studies involve follow-up into adolescence, when IQ is nearing its peak and inter-individual differences are enhanced.^16,19^ Associations of IQ in young adulthood are particularly strongly associated with socioeconomic and mortality outcomes, indicating the social and potential clinical importance of IQ in young adulthood.^26^

In addition to assessing cognition, for the first time we will also include an assessment of vision. Vision is a neurocognitive outcome that is immature at birth and programmed by visual stimuli and nutrition in early life.^27^ Inadequate infant nutrition may alter visual development,^28^ and the absence of a clear retinal image may lead to myopia.^29^ Evidence suggests that breastfeeding promotes visual development, and hence less susceptibility to myopia,^28,30-32^ findings attributed to the long-chain polyunsaturated fatty acids (LCPUFAs) present in breast milk. However, although LCPUFAs occur in high concentrations in retinal photoreceptors, trials comparing LCPUFA- supplemented with un-supplemented formula milk are equivocal about their role in visual development.^33^ Inconsistencies may be due to differences in statistical power and/or the degree of adjustment for confounders.^28,30^ An intention-to-treat analysis in PROBIT would provide robust evidence on the association of breastfeeding with visual outcomes.

**2.1.2 Atopic eczema**

Breastfeeding is considered an important strategy to prevent the development of atopic eczema and other allergic diseases.^34^ Many allergy organizations, ministries of health, and the World Health Organization recommend between four and six months of exclusive breastfeeding to aid allergy prevention.^34^ These recommendations are largely based on cross-sectional studies.^35,36^ A meta-analysis of 21 observational cohort studies found no convincing evidence for a protective effect of exclusive breastfeeding for at least 3 months on atopic eczema risk up to 4.5 years age^37^, but we are aware of no study that has assessed the impact of prolonged and exclusive breastfeeding on atopic eczema risk during adolescence or later in life.

Methodological shortcomings might explain some of the above contradictions. Observational studies are prone to confounding, in particular due to substantial differences between mothers who do and do not choose to breastfeed, making it difficult to determine whether the observational associations of breastfeeding duration with child health outcomes are causal or have alternative explanations. Observational studies may also be vulnerable to reverse causality, because a mother’s feeding behaviour (whether or not to continue breastfeeding) may be influenced by an infant’s having developed atopic eczema. That may be the reason why some observational studies have found higher atopic eczema risk with longer duration of exclusive breastfeeding.^38^

The PROBIT breastfeeding promotion intervention reduced examination-based atopic eczema incidence in infancy (cluster-adjusted OR, 0.54; 95% CI, 0.31-0.95).^21^ In contrast, no evidence of a protective effect was observed at 6.5 years of age ^50^, but that result was based on questionnaire-based outcomes, rather than physical examination. Relying on questionnaire-derived outcomes for atopic eczema has previously been shown to be inadequate for risk factor analyses, as it attenuates associations; skin examination, using validated diagnostic criteria, is the gold standard, even for large population-based studies.^39^ In this proposal, we will conduct intention-to-treat analysis of PROBIT participants by standardized skin examination, conducted by trained study pediatricians, to provide further experimental evidence on whether increased breastfeeding duration and exclusivity protects against atopic eczema diagnosed over the long term.

**2.1.3. Lung function**

Childhood asthma, a leading cause of excess emergency visits, hospitalizations and missed school, is strongly influenced by exposures in early life.^40^ At birth, the lungs are immature. The number of alveoli increases rapidly during infancy, a period when lung function and growth may be particularly sensitive to nutritional or environmental exposures.^41^ Lung function subsequently tracks along percentiles from childhood into adulthood. There is evidence that lung function shortly after birth is associated with later asthma.^42,43^ Asthma inflammation can in turn affect lung function, and although lung function may be normal between flares in individuals, at a population level asthma is associated with reduced forced expiratory volume (FEV_1_). Reduced lung function may be an intermediate phenotype of asthma, but also might also be an outcome independent of asthma status. Thus, early-life adverse exposures have been associated with lung function throughout life.^44^

A protective effect of breastfeeding on asthma has been observed,^45,46^ and attributed to various potential biological mechanisms. For example, reduced exposure to cow milk and other foreign antigens may ameliorate the atopic immune response,^47^or abundant cytokines (e.g. TGF-β1) in breastmilk may benefit lung and airways growth and development.^48^ A reduced frequency or severity of respiratory infections in infancy, which has been observed with breastfeeding in most studies,^49,50^ may, in turn, protect the development of lung function in childhood.^51^ However, some studies report an increased asthma risk with breastfeeding,^52^ particularly in older children of atopic mothers,^49,50^ in line with animal studies suggesting that breastmilk substances (e.g. IgE or interleukins) may trigger airway hyper-responsiveness and allergic inflammation.^53^ Other studies are null,^54^ and some indicate that the apparent protection of breastfeeding may arise by reverse causality, whereby early signs of atopic disease lead to earlier discontinuation of exclusive breastfeeding.^55^

In PROBIT, wheezing, atopic symptoms and asthma diagnoses were more prevalent in the experimental versus control group at 6.5 years, although the wide confidence intervals could not exclude an important protective or adverse effect. ^56^ Asthma symptoms and diagnoses were ascertained with the International Study of Asthma and Allergy in Childhood (ISAAC) questionnaire and an asthma diagnosis was rare (1%). Thus, potential measurement error and small numbers limited study power. The potential biological mechanisms outlined above imply that any link of breastfeeding with asthma may be mediated via effects on lung function during the sensitive period of lung growth in infancy. Furthermore, children with asymptomatic lung function impairment are at increased risk of chronic respiratory disease in later life,^42,57^ so lung function measures are a clinically relevant intermediate phenotype. Breastfeeding has been associated with substantially higher lung volumes in adolescence,^58,59^ with some evidence of a possible beneficial effect on airflow obstruction.^59,60^ Using spirometry, we propose to measure lung volumes (FEV_1_ and FVC) and flow rate (PEFR), objective outcomes on a continuous scale that will provide greater power to detect effects compared to binary, self-reported outcomes such as asthma symptoms and diagnosis.

**2.1.4 Adiposity and blood pressure**

The prevalence of childhood obesity has risen substantially in recent decades around the world.^61^ In turn, obese children are more likely to become obese adults^62^ and suffer obesity-related chronic illnesses.^63^ However, few interventions to prevent childhood obesity have proven effective.^61,64^ Promoting greater uptake and duration of exclusive breastfeeding is a suggested public health measure to reduce childhood obesity^65^ and its metabolic consequences (e.g. high blood pressure, BP).^66^ This is based on mechanistic studies, for example those finding that the lower protein content of breastmilk (in comparison to formula milk) may reduce adipocytes development,^67^ and a body of observational human data suggesting inverse associations of breastfeeding and its duration with later obesity.^66,68-72^ However, observational studies are prone to confounding by social patterning of both breastfeeding and growth,^73^ the epidemiological evidence is inconsistent^66,73-83^ and publication bias is a concern.^3,84^ Weight and blood pressure change dynamically during development, but most previous studies measure these outcomes on a single occasion, rather than on multiple occasions at different ages among the same individuals.

**2.1.5. Summary**

Our proposed research will provide an estimate of the causal effect of breastfeeding on important child health outcomes. Understanding the causal effects of breastfeeding is important to maximize the population-wide impact of guidelines focusing on early nutrition,^8,85-89^ by providing robust data, ensuring consistent conclusions,^85,89^ and minimizing potential harms.^90^ These results will also inform social policies to promote breastfeeding and reduce its socioeconomic inequalities. For example, the recent health care overhaul does will not reimburse women for breast pumps, as the Internal Revenue Service has ruled that breastfeeding does not have proven health benefits.^91^ Our findings may also drive further research to understand underlying mechanisms that could inform the diet of lactating women and the development of infant milk formula. For example, if future work determines that LCPUFAs explain any real long-term effects of breast milk on the studied outcomes,^92^ prenatal and infant nutrition could be optimized to include these nutrients. We focus on adiposity, blood pressure, neurocognitive and lung function at 15-16 years because these outcomes in childhood are linked to future psychological and social functioning, cardio-respiratory disease and mortality.^93-98^ Experimental data suggest a long-term benefit of breastfeeding on cognition is plausible,^12,17^ but the link and effect-size remain controversial.^13,14^ For lung function an adverse effect of breastfeeding has not been ruled out,^52^ despite decades of research.^99^ Research suggests that breastfeeding may reduce atopic eczema among infants, but there is limited evidence that this benefit extends into adolescence.^34,37,^ As we are already assessing other major child health outcomes of breastfeeding, namely metabolic and cardiovascular risk, in PROBIT III at 11.5 years, neurocognitive and lung function are the most important remaining outcomes requiring further study.

**2.2. Innovation**

The long-term follow-up of PROBIT, with intention-to-treat analysis, provides a unique opportunity to robustly test whether breastfeeding causally influences long-term adiposity, blood pressure, neurocognitive outcomes and lung function. Instrumental variable analysis will provide unconfounded estimates of the effect of breastfeeding on the outcomes. A recent meta-analysis of the effects of breastfeeding on maternal and child health noted that the breastfeeding literature is comprised primarily of observational studies.^100^ No other study of the magnitude and scope of PROBIT exists, and no prior randomized trials of breastfeeding have included outcomes past infancy.

**2.3. Approach**

**2.3.1. People and track record**

The study team unites expertise in the US, UK, Canada and the Republic of Belarus, and has already established a successful working relationship, having collaborated on the recent follow-up of PROBIT children at 11.5 years (PROBIT III). Under the leadership of Drs. Martin, Oken, and Kramer, the study team maintained the keen interest and active involvement of the participating Belarusian pediatricians and scientists, and obtained a remarkable 82% response rate for PROBIT III. For the proposed project we will employ similar strategies to ensure communication and productivity, including regular conference calls and twice-yearly joint visits to Minsk to meet with Belarusian pediatricians, research scientists and staff. Drs. Kramer, Martin and Oken have detailed knowledge of the project infrastructure in Belarus and a successful working partnership with the Minsk-based coordination centers (Ministry of Health; National “Mother and Child” Center, director Dr. Vilchuck, collaborator). We will employ this comprehensive knowledge and a network of key contacts to facilitate the project’s smooth running. We further detail the roles and expertise of study PI’s Drs. Oken and Martin, and senior co-I Dr. Kramer, in the Multiple PI leadership plan.

In addition to their work together on PROBIT, each of the study key personnel has led important research in the child health effects of early nutrition. Dr. Martin has authored several papers on the long-term effects of breastfeeding that have influenced clinical guidelines, including a series of influential meta-analyses.^2,4,5,101-103^ He is co-PI on the Boyd Orr cohort that has completed over 65 years of follow-up of children born in the 1930s, providing experience with cohort maintenance, questionnaire design and assessment and analysis of cognitive outcomes and lung function (spirometry). Dr. Oken has led or collaborated on studies and authored influential reviews and commentaries examining associations of maternal prenatal fatty acid status and other nutritional factors with child neurocognitive and immune development.^104-109^ She is co-PI of Project Viva, a longitudinal pre-birth cohort study that has followed women and children in the Boston area for over a decade. Through her work on Project Viva she has experience with questionnaire design, and assessment and analysis of child vision, cognition, and lung function via spirometry. The PI’s are thus well-qualified to lead the proposed study.

Other co-Investigators contribute extensive breadth and depth of experience. In addition to leading the first two phases of PROBIT, senior co-Investigator Dr. Kramer has had a long career investigating aspects of birth outcomes, infant feeding and infant growth. He has written about the difficulties of interpreting observational data on the association between breastfeeding and later health outcomes,^99,110^ and has authored influential papers on the association between breastfeeding and obesity.^23,111-113^ On behalf of the World Health Organization, he completed an extensive systematic review of the relationship between duration of breastfeeding and later health in childhood.^85^ Dr. Gillman is an internationally recognized expert in the developmental origins of health and disease.^114-116^ As PI of the Project Viva cohort and through his experience leading follow-up of children in the Framingham Children’s Study, the Growing Up Today Study, and the Early Determinants of Adult Health study, he has extensive experience in conducting and analyzing data from epidemiologic studies across the age spectrum.^117-120^ Professor Davey Smith is an eminent epidemiologist who led the establishment of an MRC Centre for Causal Analyses in Translational Epidemiology at the University of Bristol with a specific aim of developing methods to establish causality from population studies, including the long-term follow-up of RCTs.^3,121-123^ Dr. Henderson is a pediatric respiratory physician with extensive experience in measuring lung function in large population studies.^124,125^ Dr. Owen initially trained as an optometrist and has a longstanding interest in ophthalmic epidemiology. He has co-authored with Dr. Martin several papers examining the effect of infant feeding on health,^2-5^ and is involved in 3 UK studies measuring refractive status in children.^126-128^ Dr. Carsten Flohr is trained in both pediatrics and dermatology; his research examines how genetic, immunological and environmental factors contribute to the development of atopic eczema and other atopic conditions.^39,129,130^ Dr. Yang is a social epidemiologist with a background in psychology and a research focus on determinants of cognitive ability in children and young adults, including collaborations with PROBIT.^131-133^ Dr. Kleinman is a senior biostatistician with extensive experience analyzing data from cluster randomized trials and longitudinal cohort studies including specific expertise in analysis of repeated longitudinal measures and other clustered data, missing data problems, and Bayesian techniques.^119,134-138^ Thus, we have assembled an experienced team with a track record of productive, influential research and a long history of collaborative relationships that will ensure the successful completion of the proposed aims.

**2.3.2. Environment**

Each of the study key personnel comes from an academic setting with extensive experience in the design, conduct, and analysis of longitudinal studies of child health. The Department of Population Medicine at Harvard Medical School and Harvard Pilgrim Health Care is internationally recognized for its influential research in population health, including studies of the lifecourse influences on later health and disease risk. Bristol’s School of Social and Community Medicine demonstrates similar excellence in epidemiological research, including in early-life risk factors for chronic disease. In the 2008 UK Research Assessment Exercise, 70% of the School’s epidemiology research was rated as world leading/internationally excellent. The School houses the MRC Centre for Causal Analyses in Translational Epidemiology, which will be available to provide methodological expertise, and the Avon Longitudinal Study of Parents and Children (ALSPAC) which has provided Drs. Martin, Henderson and Davey Smith with enormous experience of detailed phenotyping in large-scale prospective birth cohorts.

**2.3.3. The PROBIT study**

**a. Study Population**

Thirty-four maternity hospitals and one each of their affiliated polyclinics (the clinics where children are followed for routine health care) were randomly assigned to receive a breastfeeding promotion intervention (experimental group) or to continue the prevailing maternity hospital and polyclinic practices at the time of randomization (control group). Cluster randomization was preferred over individual randomization because randomizing individual women within the same maternity hospital to different interventions would probably have led to contamination and a dilution of the effect of the intervention. After randomization, two hospitals from two different pairs refused to participate and a third randomized site was removed from the trial because of documented falsification of outcome data.^139^ This left 16 intervention and 15 control sites in the trial.

| ***Table 1: Baseline comparison of mothers and infants enrolled in the PROBIT Trial*** | | |
| --- | --- | --- |
| **Variable** | **Control (n=8181)** | **Experimental (n=8865)** |
| Maternal age 20-34 (%) | 82.3 | 81.4 |
| University education (%) | 13.0 | 14.1 |
| No other children in the household (%) | 56.1 | 59.8 |
| Gestational age (weeks) | 39.3 | 39.4 |
| Birth weight (g) | 3446 | 3448 |
| Birth length (cm) | 52.2 | 51.9 |
| Birth head circumference (cm) | 34.8 | 35.1 |
| Apgar score at 5 minutes (%) | 8.5 | 8.6 |
| Male sex (%) | 51.6 | 51.8 |

Recruitment for PROBIT began in June 1996 and continued until the end of December 1997. Mothers were eligible for participation if they initiated breastfeeding on admission to the postpartum ward, had no illnesses that would contraindicate breastfeeding or severely compromise its success, and had given birth to a healthy singleton infant of 37 weeks or more gestation, 2500 g or more birth weight, and Apgar score 5 or higher at 5 minutes. Study staff estimated that only 1-2% of eligible women declined participation. Since all women initiated breastfeeding, the experimental intervention was designed to increase the duration and exclusivity of breastfeeding. A total of 17,046 mothers (n=8865 intervention and n=8930 controls) had surviving infants. The randomization procedure produced groups with similar distributions of potentially confounding sociodemographic and clinical characteristics^21^ (**Table 1**)**.**

**b. The PROBIT Intervention**

***Table 2: The Ten Steps of the WHO’s Baby Friendly Hospital Initiative (BFHI).***

| 1 - | Maintain a written breastfeeding policy that is routinely communicated to all health care staff. |
| --- | --- |
| 2 - | Train all health care staff in skills necessary to implement this policy. |
| 3 - | Inform all pregnant women about the benefits and management of breastfeeding. |
| 4 - | Help mothers initiate breastfeeding within one hour of birth. |
| 5 - | Show mothers how to breastfeed and maintain lactation, even if they are separated from their infants. |
| 6 - | Give infants no food or drink other than breastmilk, unless medically indicated. |
| 7 - | Practice “rooming in”-- allow mothers and infants to remain together 24 hours a day. |
| 8 - | Encourage unrestricted breastfeeding. |
| 9 - | Give no pacifiers or artificial nipples to breastfeeding infants. |
| 10- | Foster the establishment of breastfeeding support groups and refer mothers to them on discharge. |

The experimental intervention included 10 steps that maternity hospitals must implement to become certified as ‘Baby Friendly’ (**Table 2**).^140^ Participants, usually the chief obstetrician and pediatrician from each of the intervention maternity hospitals and polyclinics, respectively, received the 18-hour Baby Friendly Hospital Initiative (BFHI) lactation management training course, which was organized by the European Regional Office of the WHO. The course emphasized methods to maintain lactation, promote exclusive and prolonged breastfeeding, and resolve common problems. Full implementation of the experimental intervention required 12 to 16 months to train all midwives, nurses, and physicians providing care to study mothers and infants during labor, delivery, and the postpartum hospital stay, and all pediatricians and nurses working at the polyclinics. Monitoring visits were conducted before and during recruitment and follow-up to ensure compliance with and maintenance of the study protocols.

**c. Data collection and validation at enrollment and in infancy**

Sociodemographic and clinical information was recorded on an enrollment form completed during the postpartum stay. In Belarus, infants are seen monthly for routine well-child visits and whenever they are ill. Classification of the degree of breastfeeding was based on WHO definitions.^140^ An infant was considered to be exclusively breastfed at three months if the feeding information obtained at one, two, and three months indicated that he or she was being breastfed and that no solids, non-breast milk, or water or other liquids (other than vitamins or medications) were being administered to the infant. An infant was considered to be exclusively breastfed at 6 months if, in addition to the above criteria, he or she was not receiving any other liquid or solid foods at the 6-month visit. An infant was considered to be predominantly breastfed if he or she received no solids or non-breast milk; juices, water, teas, and other liquids were permitted in this category.

Because the clinical outcomes were measured by the same pediatricians involved in implementing the experimental intervention or usual care, they could not be blinded to the intervention or control status of the study infants. A routine audit of data validity was therefore carried out at each study site. Twenty polyclinic charts were selected at random and the data on breastfeeding and infant outcomes at three months were compared with the data on these outcomes recorded on the PROBIT polyclinic visit forms. Of the 20 audited polyclinic charts, maternal interviews were also carried out for 10. For breastfeeding at three months, agreement was considered present if the date of weaning in the polyclinic chart or by maternal interview was within 15 days of the date recorded on the PROBIT polyclinic visit forms. For continued breastfeeding at three months, the kappa values for maternal interviews were 0.89 (95% CI: 0.81-0.97) in the experimental group and 0.94 (0.89-0.99) in the control group. Information about the outcome data collected at each PROBIT follow-up visit is summarized in **Table 3** and detailed below.

| ***Table 3. Visit schedule and previous funding for the PROBIT studies*** | | | |
| --- | --- | --- | --- |
| **PROBIT phase** | **Funding source** | **PI** | **Funded components** |
| PROBIT I  (Infant follow-up through 1 year) | Canadian Institutes of Health Research | Kramer | Baseline characteristics at enrollment  Infant growth, infectious diseases, and atopic eczema |
| PROBIT II  (Child follow-up through 6.5 years) | Canadian Institutes of Health Research | Kramer | Child adiposity, asthma, allergy, dental caries, intelligence & behavior |
| PROBIT III  (Child follow-up through 11.5 years) | Canadian Institutes of Health Research | Kramer | Child adiposity and blood pressure |
|  | European Union (EARNEST Project) | Martin | Biomarkers of growth and heart disease risk in children (IGF-I, insulin, glucose, apoA1, apoB) |
|  | NICHD (R01 HD050758) | Oken | Child adiponectin and child metabolic syndrome |
| ***PROBIT IV*** | ***Current proposal*** | ***Oken & Martin*** | ***Child cognition, vision, atopic eczema, lung function, adiposity measures and blood pressure*** |

**d. Outcomes in infancy (PROBIT I)**

The first phase of follow-up (PROBIT I) assessed intervention effects on: breastfeeding duration/exclusivity; gastrointestinal and respiratory infection; and atopic eczema among infants.^21^ As seen in **Figure 1**, infants from intervention sites were 7 times more likely to be exclusively breastfed at 3 months (43.3% vs. 6.4%; P<0.001) and 13 times more likely at 6 months (7.9% vs. 0.6%; P = 0.01). Nearly twice as many intervention women were predominantly breastfeeding at 3 months (51.9 vs. 28.3%, p < 0.05) and nearly 7 times as many at 6 months (10.6% vs. 1.6%; P = 0.003). These differences were large enough to cause an appreciable reduction in gastrointestinal tract infections (9.1% vs. 13.2%) and atopic eczema (3.3% vs. 6.3%).

**e. Outcomes at age 6.5 years (PROBIT II)**

In PROBIT II, associations of breastfeeding promotion with child outcomes, including atopic disease, cognitive development, behavior, growth, obesity and blood pressure, were investigated. When the children were approximately 6.5 years of age, pediatricians invited them to the polyclinics for a study visit lasting about 1.5 hours. The participation rate was 13,889 (85%) of the 16,442 followed-up at 12 months (82% of the original cohort of 17,046). Outcomes included atopy,^56^ behavior,^25^ growth and blood pressure,^23,113^ and dental caries.^24^ Results for the IQ and asthma outcomes are described in detail above (**Section 2.1.**).

**f. Outcomes at age 11 years (PROBIT III)**

***Table 4: Intra-class correlation coefficients (ICC) for measurements taken in PROBIT III***

| *Measurement* | *ICC* |
| --- | --- |
| Standing height | 0.03 |
| Sitting height | 0.03 |
| Weight | 0.02 |
| Bioimpedance | 0.01 |
| Mid-upper arm circumference | 0.03 |
| Systolic blood pressure | 0.12 |
| Waist circumference | 0.07 |
| Hip circumference | 0.03 |
| Head circumference | 0.10 |
| Triceps skinfold thickness | 0.12 |
| Subscapular skinfold thickness | 0.04 |
| Glucose | 0.08 |

At 11.5 years (PROBIT III), we investigated effects of breastfeeding on growth and cardio-metabolic risk factors. Pediatricians performed anthropometric measurements on mothers and children, and collected fasting blood via fingerstick. We have completed all study visits and await final data entry and results of laboratory assays. The participation rate - 13,891 children were seen – attests to our ability to maintain excellent follow-up and thus minimize retention bias. Intra-class correlation coefficients were extremely low for all measured outcomes (**Table 4**), indicating valid measurements with a low degree of clustering.

In an interim intention-to-treat analysis (n = 12,374), cluster-adjusted mean differences in experimental vs. control groups were essentially null: 0.16kg/m^2^ (95% CI: -0.08, 0.40) for BMI; 0.43% (-0.15, 1.01) for body fat; 0.33cm (-1.25, 1.92) for waist circumference; 0.11mm (-1.53, 1.74) for triceps skinfold thickness; 0.62cm (-0.40, 1.64) for height; and 1.12mmHg (-1.06, 3.30) for systolic blood pressure.^141^ The odds ratio for obesity (experimental vs. control arms) was 1.19 (0.95, 1.50). We found similar null results for maternal postpartum adiposity.^142^ We now have a number of papers in progress presenting results of the intention-to-treat analysis of the anthropometric and biochemical study outcomes. Our joint experience leading PROBIT III yields several important messages: we have demonstrated our successful collaboration in leading a PROBIT outcome visit; we achieved high rates of follow-up with little evidence for bias in follow-up or clustering in outcome assessments; and we require 4 years of funding to allow for complete data collection and analysis.

**g. Summary**

The PROBIT intervention successfully improved the duration of total and exclusive breastfeeding in a large population of mothers and infants. Participant characteristics were evenly balanced at study enrolment, suggesting that randomization was successful and confounding by measured and unmeasured characteristics is unlikely. At every phase of follow-up (I, II & III), those followed up (> 80%) had similar baseline characteristics *vs.* non-responders, providing reassurance against follow-up bias. Data collection through 11.5 years suggests high follow-up rates and uniform, high-quality outcome data, minimizing the likelihood of bias. The experienced and productive team will build upon established relationships to accomplish the proposed aims of this project.

**2.3.4. PROBIT IV (current proposal)**

**a. Eligible population**

We will attempt to follow-up all participants enrolled in PROBIT I, most of whom were seen at age 11.5 years (PROBIT III). Follow-up will commence in June 2012 when the oldest child will be 16 years. We will “front load” recruitment so that virtually all children are evaluated as near as possible to their 15^th^ birthday and as few children as possible will be of school-leaving age (17 years). We again plan to follow up 14,000 children (82% of the original cohort). Given 97% follow-up in PROBIT I and 82% follow-up in both PROBIT II and III, we feel this goal is achievable; furthermore, the follow-up protocol will be shorter (1 vs. 2 hrs) and does not involve fasting or blood sampling, so will be more convenient for both children and clinicians; and we have recently updated contact information on the large majority of children seen between 2008-2010 for PROBIT III. Emigration levels are low and few families move their residence, except within Minsk. However, it is critical that we begin data collection soon, as after age 17 many children leave home to attend university, and we may lose the opportunity for further contact.

**b. New data**

As in PROBIT I-III, polyclinic pediatricians will locate and invite children to attend the follow up clinic examination where the pediatricians will collect the measures described below.

***Informed consent****.* As we have done at each stage of data collection, pediatricians will obtain verbal informed consent via telephone or written informed consent from a parent for each participating child. They will also obtain verbal assent from children. Belarusian law disallows minors from providing written informed consent.

***Neurocognitive function.***  Cognition will be assessed using the *Mindstreams* Global Assessment Battery (NeuroTrax Corp), a software package that is installed onto PCs and contains a validated computerized cognitive battery with multiple domains.^143-145^ After detailed review of available instruments, we chose *Mindstreams* because it tests a breadth of cognitive function; is brief (≈ 45 mins); includes a verbal domain (the domain most strongly associated with breastfeeding in PROBIT II); is self-administered; has an available, validated Russian language version; requires little computer literacy (only mouse clicks and number pad entries); and includes built-in trial tests with feedback before starting, to ensure comprehension. Other available tests are longer (> 1 hour); do not include verbal ability or have existing verbal tests that may not be generalizable to Russian; or are administered by trained personnel, so are not practical/cost-effective in our large trial. The *Mindstreams* non-verbal ability domain tests reaction time, immediate and delayed non-verbal memory (geometric figures), motor skills (e.g. hand-eye coordination; spatial recognition) and visual-spatial processing. The verbal ability domain tests immediate and delayed verbal memory (words; pictures) and word recognition. Aggregate outcome parameters will be computed from raw data using automatic algorithms, blind to intervention, standardized by age and fitted to an IQ-style scale (mean 100, SD 15). Standardized parameters that measure similar cognitive functions will be averaged to produce 7 ‘index scores’, each summarizing performance in a single domain:^146^ memory; executive function; visual-spatial perception; verbal function; attention; information processing; and motor skills. A global cognitive score, reflecting general cognitive status, will be computed as the average of the index scores.

***Vision****.* We will use the LogMAR-Crowded test (<http://www.keeler.co.uk>) to measure vision without optical correction, visual acuity with optical correction (if present) and pinhole acuity in each eye, to determine if any deficit in vision is refractive. A vision cut-off (LogMAR 0.2, equivalent to worse than 6/9 Snellen acuity) will be used to define myopia; this threshold has high sensitivity (>90%) and specificity in identifying myopia,^126,147^ and that 91% of those with this level of vision at 16 years will be myopic in later life.^148^ The definition of myopia will be validated in a subset of children from the largest polyclinic (n=1000) using open field auto-refraction with a distant fixation target (Maltese cross at 5 meters) to stabilize accommodation without need for pharmaceutical cycloplegia (which could otherwise compromise participation).

***Atopic eczema.*** We will identify cases of atopic eczema through physical skin examinations conducted by the PROBIT study pediatricians, and collect additional data on eczema symptoms and skin health through questions from the International Study of Allergy and Asthma in Children (ISAAC) questionnaire. Study pediatricians will examine PROBIT participants for signs of flexural dermatitis around the eyes, the back of the neck, the front of the elbows, the back of the knees, and the front of the ankles. Evidence of flexural dermatitis upon exam at any of the 5 examination sites is sufficient for a diagnosis of atopic eczema.

***Lung function****.* We will measure peak expiratory flow rate (PEFR), forced expiratory volume in one second (FEV_1_), forced vital capacity (FVC), and FEV_1_/FVC ratio using the ‘Micro spirometer Plus’ (Micromedical, [www.micromedical.co.uk](http://www.micromedical.co.uk)), which meets American Thoracic Society (ATS) and European Respiratory Society (ERS) criteria.^149^ ATS/ERS guidelines will be followed in performing the measures and assessing acceptability and reproducibility.^150^ Room temperature and barometric pressure will be recorded. Children will be asked to complete the maneuver 5 times and the results and technical acceptability (i.e. maximum inspiration, a good start, a smooth continuous exhalation and maximal effort)^150^ of each blow recorded. We will aim for 3 acceptable and 2 repeatable (two largest values of FVC/FEV_1_ within 0.15 liters of each other) maneuvers.^150^ Pediatricians will also measure child height using existing PROBIT stadiometers as they have done previously.^23^ The child will self-complete the European Community Respiratory Health Study questionnaire,^151^ which includes measures of respiratory symptoms, self-reported asthma/allergy, smoking, indoor exposures and asthma treatment.

***Adiposity and blood pressure.*** Systolic and diastolic blood pressure readings will be measured using the semi-automated oscillometric Omron 705IT; (Omron Health Care, UK) using appropriate cuff size. The time of taking the measurement and room temperature (measured with an electronic thermometer) will be recorded. Standing height will be measured in duplicate (and averaged) using a wall-mounted. Waist circumference will be measured in duplicate at the mid-point between the iliac crest and the lower edge of the ribs in the mid-axillary line using a nonstretchable cloth tape measure. The Tanita TBF 300 GS body-fat analyzer (Tanita Corp, Tokyo, Japan) will be used to measure leg-to-leg bioimpedance from which weight, percent body fat, fat mass, and fat free mass are calculated.

**c. Training of pediatricians**

To ensure that high quality data are collected and that repeatable measurements can be obtained, we will hold a training workshop prior to starting fieldwork (as we have done in all past follow-ups). We will re-certify pediatricians in height measurement and certify them in the spirometry, cognitive and vision measures. Spirometry training will include an instruction session (Prof Henderson), followed by practical sessions to assess each pediatrician’s coaching technique and data outputs, with individual feedback. As we have done in the past (e.g. in PROBIT II for IQ) we will practice spirometry on consenting local children who are not study participants. Training for the cognitive function test will be minimal as the pediatricians only need to provide standardized, simple instructions to the children regarding how to execute the test from the software. Dr. Yang will advise on administering the cognitive function test and related analyses. Dr. Flohr will provide training in conducting the skin examinations, followed by a written, skills-based test to confirm that the pediatricians can accurately identify signs of flexural dermatitis. Dr. Owen will provide training in vision assessment, by lecture with follow-on practical and individual feedback. Subsequent workshops will be held 6 monthly to monitor follow-up, clarify procedures and re-standardize measurements.

**d. Quality control**

*Monitoring.* Once the data collection process is underway, we will initiate data monitoring visits to each polyclinic. Trained Belarusian monitors will observe pediatricians and repeat key measurements during study visits on one full day.

*Auditing.* Once data collection is complete at each polyclinic, we will schedule auditing visits in order to assess the inter-observer reproducibility, an important step given that the pediatricians are not blinded to randomization status.^23^ The Belarusian auditors (likely the same individuals who will serve as monitors) will conduct duplicate (repeat) visits on a subset of randomly chosen PROBIT IV participants. We will perform 4 audit visits at each polyclinic with a single pediatrician, and a total of 6 audit visits (3 per pediatrician) for each polyclinic with two pediatricians. The auditors will be blinded to the initial measurements. We will calculate correlation coefficients with 95% confidence intervals for the initial vs. repeat measurements.

**e. Data management**

Using standard protocols for the questionnaire, measures and data coding, polyclinic pediatricians will be responsible for data collection. We have established a robust data management system and will build on the expertise gained in earlier follow-ups of the trial. There will be a peripherally located Microsoft Access database at the Data Center in Minsk for administration of follow-up invitation letters, appointment information, and monitoring conformity with other study procedures.

Data flow will be as follows. Copies of data forms containing the results of the physical examination and the questionnaires will be transported by driver from each polyclinic on a monthly basis to the Data Center in Minsk. Carbon copies of the forms will be kept at the polyclinic in case of loss in transit. Data will be entered into a Microsoft Access database, with built in logic checks, by trained data entry staff. Spirometric and cognitive data will be downloaded onto encrypted USB sticks, transported to Minsk by the driver and automatically uploaded into the database to prevent transcription errors. On a weekly basis, the Minsk-based data coordinator will run a program to identify logical errors and missing values, with corrections/completions requested by telephone and/or mail. Each month, a copy of the database will be e-mailed to the master database in Bristol, and subsequently to the analyst in Boston, who will perform ongoing analyses of data quality (blind to trial arm). She will calculate intra-class correlations to evaluate clustering, and will feed results back to the pediatricians at workshops held every 6 months.

**f. Organization of study operations**

Study operations will mirror the procedures we have successfully applied to PROBIT III, and are detailed more fully in the **Multiple PI Leadership Plan**. Drs. Oken and Martin together will be responsible for the scientific direction and overall conduct of the project. Dr. Martin will work with the UK-based Research Coordinator (Ms. Patel) to oversee day-to-day study logistics, including importation of equipment, planning workshops, developing training materials, creation and maintenance of the study databases. Ms. Patel will communicate with the Minsk-based co-ordination center and data entry team. Dr. Oken will supervise the US-based Project Manager (Ms. Andrews) to plan and oversee the data monitoring and data auditing visits. Ms. Andrews is also an experienced statistical analyst. She will receive monthly data downloads from Minsk, which she will use to create data summaries and identify missing, implausible, or outlying values, and will communicate these potential data errors back to the data entry team in Minsk. She will be the lead analyst for all analyses of the PROBIT age 15-16 year outcome data. With Dr. Oken’s supervision, Ms. Andrews will handle all communications with the Harvard Pilgrim Health Care IRB, and will track all expenses on the project.

Drs. Oken and Martin will communicate regularly with the study leadership in Minsk, including Konstantin Vilchuk, Director of the Belarusian Maternal and Child Health Center and Scientific Director for PROBIT in Minsk, and Natalia Bogdanovich, Director of Epidemiologic Studies at the Maternal and Child Health Center and Study Coordinator for PROBIT. To minimize any potential for confusion or miscommunication, Dr. Martin will be responsible for communicating via email with Dr. Vilchuck and Dr. Bogdanovich, with the help of an experienced Russian-English interpreter as has been done to date. At each twice-yearly visit to Minsk, the entire study leadership team (Drs. Oken, Martin, Kramer, Vilchuck, and Bogdanovich) will meet as a group to discuss operational issues.

**2.3.5. Analysis Plan**

**a. Overall Analytic Plan**

Prior to formal analysis, we will construct tables, histograms, and box-and-whisker plots of the collected data to search for missing data and implausible or extreme values that may suggest error in measurement, recording or data entry. Where possible we will return to paper records to find missing data and determine if errors can be corrected. Histograms of residuals will be investigated for normality and outcomes transformed when necessary to satisfy normality and other model assumptions, e.g., constant variance or linearity of effect. Because this is a randomized trial, the primary approach will be an intention-to-treat (ITT). All analyses will allow for the clustered nature of the data.

**b. Exposure, outcomes, and covariates**

The exposure will be randomization to the breastfeeding promotion intervention or to the control group. The expected sample size is 14,000 children from 31 polyclinics.

The main neurocognitive outcomes will be the *Mindstream* scores standardized to an IQ scale (mean 100, SD 15) for memory; executive function; visual-spatial accuracy; verbal function; attention; information processing; motor skills; and a global cognitive score. Vision will be assessed using LogMAR acuity, analyzed as a continuous variable (given its more normal distribution compared to Snellen acuity measures); prevalence of reduced LogMAR visual acuity will also be considered between intervention groups. Atopic eczema outcomes will be evidence of flexural dermatitis around the eyes, the back of the neck, front of the elbows, back of the knees, or ankles, as identified by a trained PROBIT pediatrician; secondary atopic eczema outcomes will include responses to the skin questions from the ISAAC questionnaire. Respiratory function outcomes will be PEFR, FEV_1_ and FVC, each expressed as internal z-scores adjusted for height, age and sex. Adiposity outcomes will be body mass (BMI), fat mass (FMI), and fat-free mass (FFMI) indices, waist-to-height ratio and overweight as BMI between the 85th to <95th percentiles and obesity as BMI at or above the 95th percentile, based on the Centers for Disease Control and Prevention (CDC) 2000 age- and sex-specific reference data. Blood pressure outcomes as continuous measures of systolic and diastolic blood pressure.

Since the number of polyclinics randomized in PROBIT (31) was relatively small, imbalances in baseline covariates sufficient to confound the effect estimates may have occurred. We will therefore conduct a secondary analysis in which we control for any predictors of our outcomes (unadjusted P<0.1) that are also found to be associated (P<0.1) with randomization status. To date, PROBIT analyses suggest that this circumstance is unlikely to occur (see **Table 1**); no evidence of confounding by individual-level covariates was evident in PROBIT I, II or III.^17,21^ The main hospital-level potential confounders are geographic region and urban versus rural location.

**c. Statistical Analysis.**

All analyses will allow appropriately for the clustering. We will use random effects models to account for correlation within polyclinic, as is recommended for cluster randomized trials. Results will be reported as mean differences. We will derive 95% confidence intervals and Wald tests of the null hypothesis of no association between intervention groups. Effect modification will be addressed by stratification and by formally testing for statistical interaction by introducing appropriate cross-product terms. We will initially examine results stratified by sex to ensure comparability, before we consider combining results for boys and girls.

It is well known that “per-protocol” analyses of randomized trials, in which participants are grouped according to the intervention they received, rather than that to which they were randomized, may be seriously biased. It is for this reason that the primary analyses for this study will be based on intention-to-treat. However, this approach may substantially underestimate the effect of the true exposure of interest (breastfeeding exclusivity and duration), since there was not complete contrast of breastfeeding status between the randomized groups: many intervention mothers did not exclusively breastfeed for 6 months, whereas some control mothers did. There is a substantial statistical literature showing that causal effects of interventions may be validly estimated from randomized trials, allowing appropriately for non-compliance with the intervention.^152-155^ Thus, in secondary analyses, we will apply instrumental variable methods to estimate the causal effect of the difference in breastfeeding exclusivity and duration achieved between the two randomized groups on our outcomes. In this approach, we will use randomization status as the ‘instrument’ that is independent of any confounders of the exposure-outcome relationship, and is related to the outcomes only via the exposure. We will thus validly estimate the unbiased effect of breastfeeding itself on cognition, vision, and lung function. Although these analyses will address the attenuation of the effect of breastfeeding that is inevitable in intention-to-treat analyses, they are unlikely to increase the power of the study, so sample size calculations are not affected.

**2.3.6. Power**

Based on follow-up in PROBIT I-III, we expect 14,000 participants in PROBIT IV. We calculated detectable differences based on this number of subjects in 31 clusters, an intention-to-treat (ITT) analysis, 80% power, 5% significance, and used a realistic value (0.01) of the intra-cluster correlation coefficient (ICC) based on the ICCs of outcomes in PROBIT I-III. The effective sample size in PROBIT IV, allowing for the clustered design, is the total number of anticipated participants (14,000) divided by the design effect (*Deff*), where *Deff* = 1+(*n*-1)×ICC and *n* is the average number of individuals per cluster (n=14,000/31=451).^156^ Thus, the design effect for an ICC of 0.01 is 1+4.5=5.5 and the effective sample size is 2,500 children (14,000/5.5). Expected means and SDs for each outcome were taken from childhood cohorts of similar age. Mean detectable differences, assuming exposure groups are dichotomized at the midpoint, are calculated as the product of the SD of each variable and the standardized difference (i.e. 0.1 given an effective sample size of 2,500^157^). For the categorical atopic eczema outcome at the 16-year follow-up (flexural eczema on skin examination), the study has 94% power at the 5% significance level to detect a 50% reduction in atopic eczema prevalence between the two study groups, similar to PROBIT I.^21^

**Table 5** shows the mean differences that can be detected in the comparison of the two randomized arms by ITT and the corresponding effect due to breastfeeding that would have to be present to achieve those ITT differences. The breastfeeding effect is based on the difference in exclusive breastfeeding at 3 months of 43% in the intervention arm and 6% among controls, and is derived by dividing the ITT differences by 0.37=37% (the difference in exclusive breastfeeding in the 2 arms). For comparison, published differences are shown. The sample size is adequate for detecting important differences in the outcomes (**Table 5**). If we fail to find associations in the ITT analysis, the study is powered to have narrow confidence intervals (CIs) around null estimates. The 95% CIs for zero mean differences, assuming an effective sample size of 1250 in each arm ((14,000/Deff) ÷ 2) and means/SDs in Table 2, would be: cognitive score ± 1.2; FEV_1_ ± 40mls; FVC: ± 45mls; PEFR: ± 60mls/s.

***Table 5:* *Expected control group means, SDs, and differences detectable (alpha= 0.05, 80% Power)***

| **Continuous Outcomes** | **Mean** | **SD** | **Mean detectable differences: ITT** | **Breastfeeding effect to produce detectable effect** | **Differences observed in other studies** |
| --- | --- | --- | --- | --- | --- |
| Global cognitive score | 100 | 15 | 1.5 | 4.1 | 2-5^16^ |
| FEV_1_ (mls)^a^ | 3345 | 480 | 48 | 130 | 40^59^ |
| FVC (mls)^a^ | 3700 | 575 | 58 | 150 | 54-103^58,59^ |
| PEFR (mls/s)^a^ | 6200 | 800 | 80 | 200 | 180^4,59^ |
| LogMar visual acuity | 0.02^b^ | 0.2^b^ | 0.02 | 0.05 | 0.05^5-7,101,158^ |
| Systolic blood pressure (mmHg)^159^ | 99.9 | 9.6 | 1.15 | 3.11 | Meta-analysis: 1.4 Max difference: 6.5^160^ |
| Diastolic blood pressure (mmHg)^159^ | 57.0 | 6.8 | 0.82 | 2.22 | Meta-analysis: 0.5; Max differences: 3.4^161,162^ |
| Body mass index (kg/m^2^) ^c^ | 16.2 | 2.0 | 0.024 | 0.06 | 0.4^163^; 0.28-0.57^72,164^ |

^a^Data from ALSPAC, standardized for height; ^b^Northern Ireland Childhood Errors of Refraction study (personal communication); ^c^Estimates of means and SDs obtained from ALSPAC.

**2.3.7. Timeline**

As summarized in **Figure 2**, we anticipate completing all proposed work during the 4 year duration of the project. During the first 6 months we will finalize the training materials, DVD’s, and manual of procedures. We will hold our initial training workshop midway through Year 1, after which the pediatricians will commence data collection. Monitoring visits will begin a few months after the first visits are conducted. Based on experience in PROBIT III, we anticipate all 14,000 children will be seen over 2.5 years, after which we will complete the audit visits, data analysis, and manuscript writing. Although the NIH grant supporting PROBIT III operations was a 3-year grant, we required a fourth no-cost-extension year to complete data collection and analysis. Thus, we believe that 4 years is a reasonable timeline for the proposed work.

***Figure 2: Project Timeline***

**2.3.8. Strengths and limitations**.

***Significance.*** This study is significant because it will yield results that can directly influence breastfeeding promotion policies and thereby influence child health.

***Approach.*** Our proposal combines the strength of a randomized design, minimizing confounding, and high rates of follow-up, minimizing selection bias. This project thus offers a unique opportunity to determine the long-term effect of a breastfeeding promotion intervention on child neurocognitive and lung function, adiposity and blood pressure. In the PROBIT trial, we successfully randomized units of medical care (hospitals and affiliated outpatient clinics) to breastfeeding promotion or usual care, resulting in large contrasts in breastfeeding experience between the two groups. In contrast to observational epidemiologic studies, in which residual confounding remains a concern even after adjustment for identified confounders, this intention-to-treat analysis will provide un-confounded estimates of the influence of breastfeeding on maternal adiposity. Breastfeeding was ascertained at regular intervals during infancy, with definitions of exclusive breastfeeding based on strict WHO criteria,^165^ minimizing measurement error. Modern alternatives to breastfeeding form the reference exposure, providing results of contemporary relevance.

The large sample size limits potential type 2 error for effect sizes of clinical and public health importance. We already have strong evidence supporting the internal validity of the conduct of the study, which would not be guaranteed if a study were established *de novo*. The stringent random allocation procedure produced intervention and control groups with similar baseline characteristics.^21^ Measurement error will be minimized by the use of standard, calibrated equipment. Pediatricians will be rigorously trained and retrained to obtain valid, reproducible measurements. Data monitoring and the extremely low intra-class correlations in previous phases of PROBIT (**Table 4**) support the precision of the outcome data collected to date.

We will minimize potential bias in outcome assessment by using self-administered, computer-scored measures of cognitive function. Although the polyclinic pediatricians cannot not be blinded to the intervention or control status, the pattern of results in previous phases of PROBIT follow-up (i.e. no effect seen for respiratory infections in infancy or child adiposity^21,23^), do not suggest systematic bias towards better outcomes in the intervention group. The duplicate audit visits will provide additional evidence against bias due to non-blinding. Losses to follow up and incomplete data are potential sources of bias. We expect similar high rates of follow-up as we achieved in PROBIT III. We will be able to compare and account for characteristics at baseline and at earlier follow-up visits between children who do and do not present for assessment at age 15-16. Compared with observational cohorts, registered trials are less prone to problems of selective submission and publication bias. Generalizability is a concern. As the study is in Belarus, all participants are white. However, PROBIT includes an economically diverse population, and fewer than 2% of eligible mothers refused initial participation in the study. Belarus resembles Western developed countries in both basic health services and sanitary conditions. An uncontaminated water supply is ensured and monitored throughout the Republic by public health authorities, and clinics and physicians are abundant and readily accessible, even in rural areas. Therefore, we believe it will be reasonable to generalize results to children in the US.

This proposal represents good value. Previous grants have already supported recruitment, collection of baseline characteristics, and delivery of the intervention. As detailed in the budgets, the Canadian Institutes of Health Research will provide substantial co-funding (~$1.2m). We require funding only for the collection and analysis of outcome measures. In addition to the proposed aims, great potential exists for the conduct of other epidemiologic research based on earlier follow-ups and stored blood, including genetic analyses.

***Innovation.*** This study will fill a crucial gap in the literature linking breastfeeding with important health outcomes in adolescence. Most previous studies have been observational. Additional limitations of the available literature include short duration of follow-up, recalled breastfeeding history, and assessment of asthma outcomes via questionnaire rather than objective measures such as spirometry. We will determine the causal effects of breastfeeding in the largest RCT in the field of human lactation ever, and by adding these 3 outcomes will complete analyses on a large range of outcomes over the 4 phases of PROBIT.

***Investigators and environment.*** We have assembled an outstanding team of investigators with appropriate expertise and proven collaboration and productivity. We build upon an innovative cluster randomized controlled trial that successfully increased breastfeeding duration and exclusivity. The high quality of data collection is established. The environments will provide strong support to ensure the success of the study.

**References**

1. Galson SK. The 25th anniversary of the Surgeon General's Workshop on Breastfeeding and Human Lactation: the status of breastfeeding today. *Public Health Rep.* 2009;124(3):356-358.

2. Owen CG, Martin RM, Whincup PH, Davey-Smith G, Gillman MW, Cook DG. The effect of breastfeeding on mean body mass index throughout life: a quantitative review of published and unpublished observational evidence. *Am J Clin Nutr.* 2005;82(6):1298-1307.

3. Owen CG, Martin RM, Whincup PH, Smith GD, Cook DG. Effect of infant feeding on the risk of obesity across the life course: a quantitative review of published evidence. *Pediatrics.* 2005;115(5):1367-1377.

4. Owen CG, Martin RM, Whincup PH, Smith GD, Cook DG. Does breastfeeding influence risk of type 2 diabetes in later life? A quantitative analysis of published evidence. *Am J Clin Nutr.* 2006;84(5):1043-1054.

5. Owen CG, Whincup PH, Kaye SJ, et al. Does initial breastfeeding lead to lower blood cholesterol in adult life? A quantitative review of the evidence. *Am J Clin Nutr.* 2008;88(2):305-314.

6. Martin RM, Davey Smith G. Does having been breastfed in infancy influence lipid profile in later life?: a review of the literature. *Adv Exp Med Biol.* 2009;646:41-50.

7. Martin RM, Davey Smith G, Mangtani P, Tilling K, Frankel S, Gunnell D. Breastfeeding and cardiovascular mortality: the Boyd Orr cohort and a systematic review with meta-analysis. *Eur Heart J.* 2004;25(9):778-786.

8. Ip S, Chung M, Raman G, Trikalinos TA, Lau J. A summary of the Agency for Healthcare Research and Quality's evidence report on breastfeeding in developed countries. *Breastfeed Med.* 2009;4 Suppl 1:S17-30.

9. Beck LF, Morrow B, Lipscomb LE, et al. Prevalence of selected maternal behaviors and experiences, Pregnancy Risk Assessment Monitoring System (PRAMS), 1999. *Morbidity & Mortality Weekly Report Surveillance Summaries.* 2002;51(2):1-27.

10. Pesa JA, Shelton MM. Health-enhancing behaviors correlated with breastfeeding among a national sample of mothers. *Public Health Nurs.* 1999;16(2):120-124.

11. Dobbing J, Sands J. Quantitative growth and development of human brain. *Arch Dis Child.* 1973;48(10):757-767.

12. Lucas A, Morley R, Cole TJ. Randomised trial of early diet in preterm babies and later intelligence quotient. *BMJ.* 1998;317(7171):1481-1487.

13. Der G, Batty GD, Deary IJ. Results from the PROBIT breastfeeding trial may have been overinterpreted. *Arch Gen Psychiatry.* 2008;65(12):1456-1457; author reply 1458-1459.

14. Kramer MS, Platt RW. Letter--Reply. *Arch Gen Psychiatry.* 2008;65:1458-1459.

15. Morgane PJ, Austin-LaFrance R, Bronzino J, et al. Prenatal malnutrition and development of the brain. *Neurosci Biobehav Rev.* 1993;17(1):91-128.

16. Anderson JW, Johnstone BM, Remley DT. Breast-feeding and cognitive development: a meta-analysis. *Am J Clin Nutr.* 1999;70(4):525-535.

17. Kramer MS, Aboud F, Mironova E, et al. Breastfeeding and child cognitive development: new evidence from a large randomized trial. *Arch Gen Psychiatry.* 2008;65(5):578-584.

18. Lucas A, Morley R, Cole TJ, Lister G, Leeson-Payne C. Breast milk and subsequent intelligence quotient in children born preterm. *Lancet.* 1992;339(8788):261-264.

19. Der G, Batty GD, Deary IJ. Effect of breast feeding on intelligence in children: prospective study, sibling pairs analysis, and meta-analysis. *BMJ.* 2006;333(7575):945.

20. Angelsen NK, Vik T, Jacobsen G, Bakketeig LS. Breast feeding and cognitive development at age 1 and 5 years. *Arch Dis Child.* 2001;85(3):183-188.

21. Kramer MS, Chalmers B, Hodnett ED, et al. Promotion of Breastfeeding Intervention Trial (PROBIT): a randomized trial in the Republic of Belarus. *JAMA.* 2001;285(4):413-420.

22. Kramer MS, Martin RM, Sterne JA, Shapiro S, Dahhou M, Platt RW. The double jeopardy of clustered measurement and cluster randomisation. *BMJ.* 2009;339:b2900.

23. Kramer MS, Matush L, Vanilovich I, et al. Effects of prolonged and exclusive breastfeeding on child height, weight, adiposity, and blood pressure at age 6.5 y: evidence from a large randomized trial. *Am J Clin Nutr.* 2007;86(6):1717-1721.

24. Kramer MS, Vanilovich I, Matush L, et al. The effect of prolonged and exclusive breast-feeding on dental caries in early school-age children. New evidence from a large randomized trial. *Caries Res.* 2007;41(6):484-488.

25. Kramer MS, Fombonne E, Igumnov S, et al. Effects of prolonged and exclusive breastfeeding on child behavior and maternal adjustment: evidence from a large, randomized trial. *Pediatrics.* 2008;121(3):e435-440.

26. Kajantie E, Raikkonen K, Henriksson M, et al. Childhood socioeconomic status modifies the association between intellectual abilities at age 20 and mortality in later life. *J Epidemiol Community Health.* 2010.

27. Hoffman DR, Boettcher JA, Diersen-Schade DA. Toward optimizing vision and cognition in term infants by dietary docosahexaenoic and arachidonic acid supplementation: a review of randomized controlled trials. *Prostaglandins Leukot Essent Fatty Acids.* 2009;81(2-3):151-158.

28. Chong YS, Liang Y, Tan D, Gazzard G, Stone RA, Saw SM. Association between breastfeeding and likelihood of myopia in children. *Jama.* 2005;293(24):3001-3002.

29. Wallman J. Nature and nurture of myopia. *Nature.* 1994;371(6494):201-202.

30. Williams C, Birch EE, Emmett PM, Northstone K. Stereoacuity at age 3.5 y in children born full-term is associated with prenatal and postnatal dietary factors: a report from a population-based cohort study. *Am J Clin Nutr.* 2001;73(2):316-322.

31. Singhal A, Morley R, Cole TJ, et al. Infant nutrition and stereoacuity at age 4-6 y. *Am J Clin Nutr.* 2007;85(1):152-159.

32. Sham WK, Dirani M, Chong YS, et al. Breastfeeding and association with refractive error in young Singapore Chinese children. *Eye (Lond).* 2010;24(5):875-880.

33. SanGiovanni JP, Berkey CS, Dwyer JT, Colditz GA. Dietary essential fatty acids, long-chain polyunsaturated fatty acids, and visual resolution acuity in healthy fullterm infants: a systematic review. *Early Hum Dev.* 2000;57(3):165-188.

34. Fewtrell M, Wilson DC, Booth I, Lucas A. Six months of exclusive breast feeding: how good is the evidence? *BMJ: British medical journal.* 2011;342.

35. Fergusson D, Horwood L. Early solid food diet and eczema in childhood: a 10‐year longitudinal study. *Pediatric allergy and immunology.* 1994;5(S5):44-47.

36. Forsyth JS, Ogston SA, Clark A, Florey C, Howie PW. Relation between early introduction of solid food to infants and their weight and illnesses during the first two years of life. *Bmj.* 1993;306(6892):1572-1576.

37. Yang Y, Tsai C, Lu C. Exclusive breastfeeding and incident atopic dermatitis in childhood: a systematic review and meta‐analysis of prospective cohort studies. *British journal of dermatology.* 2009;161(2):373-383.

38. Flohr C, Nagel G, Weinmayr G, Kleiner A, Strachan D, Williams H. Lack of evidence for a protective effect of prolonged breastfeeding on childhood eczema: lessons from the International Study of Asthma and Allergies in Childhood (ISAAC) Phase Two. *British journal of dermatology.* 2011;165(6):1280-1289.

39. Flohr C, Weinmayr G, Addo‐Yobo E, et al. How well do questionnaires perform compared with physical examination in detecting flexural eczema? Findings from the International Study of Asthma and Allergies in Childhood (ISAAC) Phase Two. *British journal of dermatology.* 2009;161(4):846-853.

40. Eder W, Ege MJ, von Mutius E. The asthma epidemic. *N Engl J Med.* 2006;355(21):2226-2235.

41. Calogero C, Sly PD. Developmental physiology: lung function during growth and development from birth to old age. In: Frey U, Merkus PJ, eds. *Paediatric Lung Function*. 47th ed. London: European Respiratory Society; 2010.

42. Haland G, Carlsen KC, Sandvik L, et al. Reduced lung function at birth and the risk of asthma at 10 years of age. *N Engl J Med.* 2006;355(16):1682-1689.

43. Leary S, Davey Smith G, Ness A. Smoking during pregnancy and components of stature in offspring. *Am J Hum Biol.* 2006;18(4):502-512.

44. Svanes C, Sunyer J, Plana E, et al. Early life origins of chronic obstructive pulmonary disease. *Thorax.* 2010;65(1):14-20.

45. Gdalevich M, Mimouni D, Mimouni M. Breast-feeding and the risk of bronchial asthma in childhood: a systematic review with meta-analysis of prospective studies. *J Pediatr.* 2001;139(2):261-266.

46. Oddy WH, Peat JK. Breastfeeding, asthma, and atopic disease: an epidemiological review of the literature. *J Hum Lact.* 2003;19(3):250-261; quiz 262-256.

47. Pabst HF, Spady DW, Pilarski LM, Carson MM, Beeler JA, Krezolek MP. Differential modulation of the immune response by breast- or formula-feeding of infants. *Acta Paediatr.* 1997;86(12):1291-1297.

48. Oddy WH, Halonen M, Martinez FD, et al. TGF-beta in human milk is associated with wheeze in infancy. *J Allergy Clin Immunol.* 2003;112(4):723-728.

49. Wright AL, Holberg CJ, Taussig LM, Martinez FD. Factors influencing the relation of infant feeding to asthma and recurrent wheeze in childhood. *Thorax.* 2001;56(3):192-197.

50. Matheson MC, Erbas B, Balasuriya A, et al. Breast-feeding and atopic disease: a cohort study from childhood to middle age. *J Allergy Clin Immunol.* 2007;120(5):1051-1057.

51. Tennant PW, Gibson GJ, Pearce MS. Lifecourse predictors of adult respiratory function: results from the Newcastle Thousand Families Study. *Thorax.* 2008;63(9):823-830.

52. Sears MR, Greene JM, Willan AR, et al. Long-term relation between breastfeeding and development of atopy and asthma in children and young adults: a longitudinal study. *Lancet.* 2002;360(9337):901-907.

53. Leme AS, Hubeau C, Xiang Y, et al. Role of breast milk in a mouse model of maternal transmission of asthma susceptibility. *J Immunol.* 2006;176(2):762-769.

54. Burgess SW, Dakin CJ, O'Callaghan MJ. Breastfeeding does not increase the risk of asthma at 14 years. *Pediatrics.* 2006;117(4):e787-792.

55. Lowe AJ, Carlin JB, Bennett CM, et al. Atopic disease and breast-feeding--cause or consequence? *J Allergy Clin Immunol.* 2006;117(3):682-687.

56. Kramer MS, Matush L, Vanilovich I, et al. Effect of prolonged and exclusive breast feeding on risk of allergy and asthma: cluster randomised trial. *BMJ.* 2007;335(7624):815.

57. Rudnicka AR, Owen CG, Richards M, Wadsworth ME, Strachan DP. Effect of breastfeeding and sociodemographic factors on visual outcome in childhood and adolescence. *Am J Clin Nutr.* 2008;87(5):1392-1399.

58. Guilbert TW, Stern DA, Morgan WJ, Martinez FD, Wright AL. Effect of breastfeeding on lung function in childhood and modulation by maternal asthma and atopy. *Am J Respir Crit Care Med.* 2007;176(9):843-848.

59. Ogbuanu IU, Karmaus W, Arshad SH, Kurukulaaratchy RJ, Ewart S. Effect of breastfeeding duration on lung function at age 10 years: a prospective birth cohort study. *Thorax.* 2009;64(1):62-66.

60. Nagel G, Buchele G, Weinmayr G, et al. Effect of breastfeeding on asthma, lung function and bronchial hyperreactivity in ISAAC Phase II. *Eur Respir J.* 2009;33(5):993-1002.

61. Lobstein T, Jackson-Leach R, Moodie ML, et al. Child and adolescent obesity: part of a bigger picture. *The Lancet.* 2015;385(9986):2510-2520.

62. Simmonds M, Llewellyn A, Owen C, Woolacott N. Predicting adult obesity from childhood obesity: a systematic review and meta‐analysis. *Obesity reviews.* 2016;17(2):95-107.

63. Kelsey MM, Zaepfel A, Bjornstad P, Nadeau KJ. Age-Related Consequences of Childhood Obesity. *Gerontology.* 2014;60(3):222-228.

64. Ng M, Fleming T, Robinson M, et al. Global, regional, and national prevalence of overweight and obesity in children and adults during 1980–2013: a systematic analysis for the Global Burden of Disease Study 2013. *The Lancet.* 2014;384(9945):766-781.

65. World Health Organization Commission on Ending Childhood Obesity. Final report of the Commission on Ending Childhood Obesity, World Health Organization, Geneva (2016) <http://www.who.int/end-childhood-obesity/news/launch-final-report/en/> (accessed January 26, 2016). 2016.

66. Horta BL, Loret de Mola C, Victora CG. Long‐term consequences of breastfeeding on cholesterol, obesity, systolic blood pressure and type 2 diabetes: a systematic review and meta‐analysis. *Acta Paediatrica.* 2015;104(S467):30-37.

67. Arenz S, Ruckerl R, Koletzko B, von Kries R. Breast-feeding and childhood obesity-a systematic review. *International Journal of Obesity & Related Metabolic Disorders: Journal of the International Association for the Study of Obesity.* 2004;28(10):1247-1256.

68. Weng SF, Redsell SA, Swift JA, Yang M, Glazebrook CP. Systematic review and meta-analyses of risk factors for childhood overweight identifiable during infancy. *Archives of Disease in Childhood.* 2012;97(12):1019-1026.

69. Yan J, Liu L, Zhu Y, Huang G, Wang PP. The association between breastfeeding and childhood obesity: a meta-analysis. *BMC Public Health.* 2014;14(1):1.

70. Li C, Goran MI, Kaur H, Nollen N, Ahluwalia JS. Developmental Trajectories of Overweight During Childhood: Role of Early Life Factors. *Obesity.* 2007;15(3):760-771.

71. Grube MM, von der Lippe E, Schlaud M, Brettschneider A-K. Does breastfeeding help to reduce the risk of childhood overweight and obesity? A propensity score analysis of data from the KiGGS study. *PloS one.* 2015;10(3):e0122534.

72. Gillman MW, Rifas-Shiman SL, Camargo, Jr CA, et al. Risk of overweight among adolescents who were breastfed as infants. *JAMA.* 2001;285(19):2461-2467.

73. Brion M-JA, Lawlor DA, Matijasevich A, et al. What are the causal effects of breastfeeding on IQ, obesity and blood pressure? Evidence from comparing high-income with middle-income cohorts. *International Journal of Epidemiology.* 2011:10.1093/ije/dyr1020.

74. Hancox R, Stewart A, Braithwaite I, Beasley R, Murphy R, Mitchell E. Association between breastfeeding and body mass index at age 6–7 years in an international survey. *Pediatric obesity.* 2015;10(4):283-287.

75. Fall CH, Borja JB, Osmond C, et al. Infant-feeding patterns and cardiovascular risk factors in young adulthood: data from five cohorts in low-and middle-income countries. *International journal of epidemiology.* 2011;40(1):47-62.

76. Zheng J-S, Liu H, Li J, et al. Exclusive breastfeeding is inversely associated with risk of childhood overweight in a large chinese cohort. *The Journal of nutrition.* 2014;144(9):1454-1459.

77. Estévez-González M, del Pino AS, Henríquez-Sánchez P, Peña-Quintana L, Saavedra-Santana P. Breastfeeding during the first 6 months of life, adiposity rebound and overweight/obesity at 8 years of age. *International Journal of Obesity.* 2016;40(1):10-13.

78. Van der Willik EM, Vrijkotte TG, Altenburg TM, Gademan MG, Kist-van Holthe J. Exclusively breastfed overweight infants are at the same risk of childhood overweight as formula fed overweight infants. *Archives of Disease in Childhood.* 2015;100:(10):932-937.

79. Victora CG, Barros F, Lima RC, Horta BL, Wells J. Anthropometry and body composition of 18 year old men according to duration of breast feeding: birth cohort study from Brazil. *Bmj.* 2003;327(7420):901.

80. Durmuş B, Heppe DH, Gishti O, et al. General and abdominal fat outcomes in school-age children associated with infant breastfeeding patterns. *The American journal of clinical nutrition.* 2014;99(6):1351-1358.

81. Martin RM, Gunnell D, Smith GD. Breastfeeding in infancy and blood pressure in later life: systematic review and meta-analysis. *American Journal of Epidemiology.* 2005;161(1):15-26.

82. Lawlor D, Riddoch C, Page A, et al. Infant feeding and components of the metabolic syndrome: findings from the European Youth Heart Study. *Archives of disease in childhood.* 2005;90(6):582-588.

83. Victora CG, Bahl R, Barros AJ, et al. Breastfeeding in the 21st century: epidemiology, mechanisms, and lifelong effect. *The Lancet.* 2016;387(10017):475-490.

84. Owen CG, Whincup PH, Gilg JA, Cook DG. Effect of breast feeding in infancy on blood pressure in later life: systematic review and meta-analysis. *BMJ.* 2003;327(7425):1189-1195.

85. Kramer MS, Kakuma R. *The optimal duration of exclusive breastfeeding.* Geneva: World Health Organization; 2002.

86. Horta BL, Bahl R, Martines JC, Victora CG. *Evidence of the long-term effects of breastfeeding; Systematic reviews and meta-analyses.* Geneva: World Health Organisation; 2007.

87. Clayton PE, Cianfarani S, Czernichow P, Johannsson G, Rapaport R, Rogol A. Management of the child born small for gestational age through to adulthood: a consensus statement of the International Societies of Pediatric Endocrinology and the Growth Hormone Research Society. *J Clin Endocrinol Metab.* 2007;92(3):804-810.

88. Barlow SE. Expert committee recommendations regarding the prevention, assessment, and treatment of child and adolescent overweight and obesity: summary report. *Pediatrics.* 2007;120 Suppl 4:S164-192.

89. Host A, Koletzko B, Dreborg S, et al. Dietary products used in infants for treatment and prevention of food allergy. Joint Statement of the European Society for Paediatric Allergology and Clinical Immunology (ESPACI) Committee on Hypoallergenic Formulas and the European Society for Paediatric Gastroenterology, Hepatology and Nutrition (ESPGHAN) Committee on Nutrition. *Arch Dis Child.* 1999;81(1):80-84.

90. Kennedy K, Ross S, Isaacs EB, et al. The 10-year follow-up of a randomised trial of long-chain polyunsaturated fatty acid supplementation in preterm infants: effects on growth and blood pressure. *Arch Dis Child.* 2010;95(8):588-595.

91. Kocieniewski D. Acne Cream? Tax-sheltered. Breast Pump? No. *The New York Times.* October 27, 2010.

92. Koletzko B, Lien E, Agostoni C, et al. The roles of long-chain polyunsaturated fatty acids in pregnancy, lactation and infancy: review of current knowledge and consensus recommendations. *J Perinat Med.* 2008;36(1):5-14.

93. Batty GD, Der G, Macintyre S, Deary IJ. Does IQ explain socioeconomic inequalities in health? Evidence from a population based cohort study in the west of Scotland. *BMJ.* 2006;332(7541):580-584.

94. Whalley LJ, Deary IJ. Longitudinal cohort study of childhood IQ and survival up to age 76. *Bmj.* 2001;322(7290):819.

95. Richards M, Black S, Mishra G, Gale CR, Deary IJ, Batty DG. IQ in childhood and the metabolic syndrome in middle age: Extended follow-up of the 1946 British Birth Cohort Study. . *Intelligence.* 2010;[Epub ahead of print].

96. Batty GD, Deary IJ, Tengstrom A, Rasmussen F. IQ in early adulthood and later risk of death by homicide: cohort study of 1 million men. *Br J Psychiatry.* 2008;193(6):461-465.

97. Griffith KA, Sherrill DL, Siegel EM, Manolio TA, Bonekat HW, Enright PL. Predictors of loss of lung function in the elderly: the Cardiovascular Health Study. *Am J Respir Crit Care Med.* 2001;163(1):61-68.

98. Mackenbach JP, Stirbu I, Roskam AJ, et al. Socioeconomic inequalities in health in 22 European countries. *N Engl J Med.* 2008;358(23):2468-2481.

99. Kramer MS. Does breast feeding help protect against atopic disease? Biology, methodology, and a golden jubilee of controversy. *J Pediatr.* 1988;112(2):181-190.

100. Ip S, Chung M, Raman G, et al. Breastfeeding and Maternal and Infant Health Outcomes in Developed Countries *Evidence report/technology assessment.* 2007(153):1-186.

101. Martin RM, Gunnell D, Smith GD. Breastfeeding in infancy and blood pressure in later life: systematic review and meta-analysis. *Am J Epidemiol.* 2005;161(1):15-26.

102. Martin RM, Middleton N, Gunnell D, Owen CG, Smith GD. Breast-feeding and cancer: the Boyd Orr cohort and a systematic review with meta-analysis. *J Natl Cancer Inst.* 2005;97(19):1446-1457.

103. Owen CG, Whincup PH, Odoki K, Gilg JA, Cook DG. Infant feeding and blood cholesterol: a study in adolescents and a systematic review. *Pediatrics.* 2002;110(3):597-608.

104. Oken E, Wright RO, Kleinman KP, et al. Maternal fish consumption, hair mercury, and infant cognition in a U.S. Cohort. *Environ Health Perspect.* 2005;113(10):1376-1380.

105. Oken E, Osterdal ML, Gillman MW, et al. Associations of maternal fish intake during pregnancy and breastfeeding duration with attainment of developmental milestones in early childhood: a study from the Danish National Birth Cohort. *Am J Clin Nutr.* 2008;88(3):789-796.

106. Oken E, Radesky JS, Wright RO, et al. Maternal fish intake during pregnancy, blood mercury levels, and child cognition at age 3 years in a US cohort. *Am J Epidemiol.* 2008.

107. Gold DR, Willwerth BM, Tantisira KG, et al. Associations of cord blood fatty acids with lymphocyte proliferation, IL-13, and IFN-gamma. *J Allergy Clin Immunol.* 2006;117(4):931-938.

108. Oken E, Belfort MB. Fish, fish oil, and pregnancy. *JAMA.* 2010;304(15):1717-1718.

109. Oken E. Fish intake and mercury levels: only part of the picture. *J Pediatr.* 2010;157(1):10-12.

110. Kramer MS, Guo T, Platt RW, et al. Breastfeeding and infant growth: biology or bias? *Pediatrics.* 2002;110(2 Pt 1):343-347.

111. Kramer MS, Barr RG, Leduc DG, Boisjoly C, McVey-White L, Pless IB. Determinants of weight and adiposity in the first year of life. *Journal of Pediatrics.* 1985;106(1):10-14.

112. Kramer MS, Barr RG, Leduc DG, Boisjoly C, Pless IB. Infant determinants of childhood weight and adiposity. *J Pediatr.* 1985;107(1):104-107.

113. Kramer MS, Matush L, Vanilovich I, et al. A randomized breast-feeding promotion intervention did not reduce child obesity in Belarus. *J Nutr.* 2009;139(2):417S-421S.

114. Gillman MW. Epidemiological challenges in studying the fetal origins of adult chronic disease. *International Journal of Epidemiology.* 2002;31(2):294-299.

115. Gillman MW. Lifecourse Approach to Obesity. In: Kuh D, Ben-Shlomo Y, eds. *Life Course Approach to Chronic Disease Epidemiology*. 2nd ed. London: Oxford University Press; 2004.

116. Gillman MW. Developmental origins of health and disease. *N Engl J Med.* 2005;353(17):1848-1850.

117. Gillman MW, Rifas-Shiman SL, Camargo CA, Jr., et al. Risk of overweight among adolescents who were breastfed as infants. *JAMA.* 2001;285(19):2461-2467.

118. Gillman MW, Oliveria SA, Moore LL, Ellison RC. Inverse association of dietary calcium with systolic blood pressure in young children. *Jama.* 1992;267(17):2340-2343.

119. Gillman MW, Kleinman K. Antecedents of obesity - analysis, interpretation, and use of longitudinal data. *Am J Epidemiol.* 2007;166(1):14-16; author reply 17-18.

120. Gillman MW, Rifas-Shiman SL, Kleinman K, Oken E, Rich-Edwards JW, Taveras EM. Developmental origins of childhood overweight: potential public health impact. *Obesity (Silver Spring).* 2008;16(7):1651-1656.

121. Alati R, Macleod J, Hickman M, et al. Intrauterine exposure to alcohol and tobacco use and childhood IQ: findings from a parental-offspring comparison within the Avon Longitudinal Study of Parents and Children. *Pediatr Res.* 2008;64(6):659-666.

122. Galobardes B, McCarron P, Jeffreys M, Davey Smith G. Association between early life history of respiratory disease and morbidity and mortality in adulthood. *Thorax.* 2008;63(5):423-429.

123. Kinra S, Rameshwar Sarma KV, Ghafoorunissa, et al. Effect of integration of supplemental nutrition with public health programmes in pregnancy and early childhood on cardiovascular risk in rural Indian adolescents: long term follow-up of Hyderabad nutrition trial. *Bmj.* 2008;337:a605.

124. Henderson J, Granell R, Heron J, et al. Associations of wheezing phenotypes in the first 6 years of life with atopy, lung function and airway responsiveness in mid-childhood. *Thorax.* 2008;63(11):974-980.

125. Cookson H, Granell R, Joinson C, Ben-Shlomo Y, Henderson AJ. Mothers' anxiety during pregnancy is associated with asthma in their children. *J Allergy Clin Immunol.* 2009;123(4):847-853 e811.

126. Rudnicka AR, Owen CG, Nightingale CM, Cook D, Whincup P. Ethnic differences in the prevalence of myopia and ocular biometry in 10-11 year old children: the Child Heart And Health Study in England (CHASE). *Invest Ophthalmol Vis Sci.* 2010.

127. O'Donoghue L, McClelland JF, Logan NS, Rudnicka AR, Owen CG, Saunders KJ. Refractive error and visual impairment in school children in Northern Ireland. *Br J Ophthalmol.* 2010;94(9):1155-1159.

128. O'Donoghue L, Saunders KJ, McClelland JF, et al. Sampling and measurement methods for a study of childhood refractive error in a UK population. *Br J Ophthalmol.* 2010;94(9):1150-1154.

129. Flohr C, Weiland SK, Weinmayr G, et al. The role of atopic sensitization in flexural eczema: findings from the International Study of Asthma and Allergies in Childhood Phase Two. *Journal of allergy and clinical immunology.* 2008;121(1):141-147. e144.

130. Schmitt J, Spuls P, Boers M, et al. Towards global consensus on outcome measures for atopic eczema research: results of the HOME II meeting. *Allergy.* 2012;67(9):1111-1117.

131. Yang S, Bergvall N, Cnattingius S, Kramer MS. Gestational age differences in health and development among young Swedish men born at term. *Int J Epidemiol.* 2010.

132. Yang S, Platt RW, Kramer MS. Variation in child cognitive ability by week of gestation among healthy term births. *Am J Epidemiol.* 2010;171(4):399-406.

133. Yang S, Lynch J, Susser ES, Lawlor DA. Birth weight and cognitive ability in childhood among siblings and nonsiblings. *Pediatrics.* 2008;122(2):e350-358.

134. Kleinman KP, Oken E, Radesky JS, Rich-Edwards JW, Peterson KE, Gillman MW. How should gestational weight gain be assessed? A comparison of existing methods and a novel method, area under the weight gain curve. *Int J Epidemiol.* 2007;36(6):1275-1282.

135. Kleinman KP, Ibrahim JG. A semi-parametric Bayesian approach to generalized linear mixed models. *Stat Med.* 1998;17(22):2579-2596.

136. Kleinman KP, Ibrahim JG. A semiparametric Bayesian approach to the random effects model. *Biometrics.* 1998;54(3):921-938.

137. Kleinman KP, Ibrahim JG, Laird NM. A Bayesian framework for intent-to-treat analysis with missing data. *Biometrics.* 1998;54(1):265-278.

138. Oken E, Kleinman KP, Belfort MB, Hammitt JK, Gillman MW. Associations of gestational weight gain with short- and longer-term maternal and child health outcomes. *Am J Epidemiol.* 2009;170(2):173-180.

139. Kramer MS, Chalmers B, Hodnett ED, et al. Promotion of breastfeeding intervention trial (PROBIT): a cluster-randomized trial in the Republic of Belarus. Design, follow-up, and data validation. *Adv Exp Med Biol.* 2000;478:327-345.

140. WHO/UNICEF. *Protecting, Promoting and Supporting Breastfeeding: The Special Role of Maternity Services.* Geneva, Switzerland: World Health Organization; 1989.

141. Martin RM, Patel P, Kramer M, et al. Effects of prolonged and exclusive breastfeeding on adiposity, stature and blood pressure in children aged 11.5 years: evidence from the Promotion of Breastfeeding Intervention Trial (abstract). *EARNEST scientific meeting, Munich Germany.* 2010.

142. Oken E, Guthrie L, Patel R, et al. Effects of prolonged and exclusive breastfeeding on maternal adiposity and blood pressure at 11.5 years postpartum: evidence from the Promotion of Breastfeeding Intervention Trial (PROBIT), a cluster-randomized controlled trial (abstract). *EARNEST scientific meeting, Munich Germany.* 2010.

143. Schweiger A, Abramovitch A, Doniger GM, Simon ES. A clinical construct validity study of a novel computerized battery for the diagnosis of ADHD in young adults. *J Clin Exp Neuropsychol.* 2007;29(1):100-111.

144. Dwolatzky T, Whitehead V, Doniger GM, et al. Validity of a novel computerized cognitive battery for mild cognitive impairment. *BMC Geriatr.* 2003;3:4.

145. Dwolatzky T, Whitehead V, Doniger GM, et al. Validity of the Mindstreams computerized cognitive battery for mild cognitive impairment. *J Mol Neurosci.* 2004;24(1):33-44.

146. Lavi R, Doniger GM, Simon E, Hochner-Celnikier D, Zimran A, Elstein D. The effect of hormone replacement therapy on cognitive function in post-menopausal women. *QJM.* 2007;100(9):567-573.

147. Leone JF, Mitchell P, Morgan IG, Kifley A, Rose KA. Use of visual acuity to screen for significant refractive errors in adolescents: is it reliable? *Arch Ophthalmol.* 2010;128(7):894-899.

148. Cumberland PM, Peckham CS, Rahi JS. Inferring myopia over the lifecourse from uncorrected distance visual acuity in childhood. *Br J Ophthalmol.* 2007;91(2):151-153.

149. Dirksen A, Madsen F, Pedersen OF, Vedel AM, Kok-Jensen A. Long-term performance of a hand held spirometer. *Thorax.* 1996;51(10):973-976.

150. Miller MR, Hankinson J, Brusasco V, et al. Standardisation of spirometry. *Eur Respir J.* 2005;26(2):319-338.

151. Janson C, Anto J, Burney P, et al. The European Community Respiratory Health Survey: what are the main results so far? European Community Respiratory Health Survey II. *Eur Respir J.* 2001;18(3):598-611.

152. Robins JM, Tsiatis AA. Correcting for non-compliance in randomized trials using rank-preserving structural failure time models. . *Communications in Statistics.* 1991;20:2609-2631.

153. Angrist JD, Imbens GW, Rubin DB. Identification of causal effects using instrumental variables. *J Amer Statist Assoc.* 1996;91:444-472.

154. Cuzick J, Edwards R, Segnan N. Adjusting for non-compliance and contamination in randomized clinical trials. *Stat Med.* 1997;16(9):1017-1029.

155. Frangakis CE, Rubin DB, Zhou XH. Clustered encouragement designs with individual noncompliance: bayesian inference with randomization, and application to advance directive forms. *Biostatistics.* 2002;3(2):147-164.

156. Ukoumunne OC, Gulliford MC, Chinn S, Sterne JA, Burney PG, Donner A. Methods in health service research. Evaluation of health interventions at area and organisation level. *BMJ.* 1999;319(7206):376-379.

157. Altman DG. *Practical Statistics for Medical Research.* 1st ed. London: Chapman & Hall; 1991.

158. Birch EE, Garfield S, Castaneda Y, Hughbanks-Wheaton D, Uauy R, Hoffman D. Visual acuity and cognitive outcomes at 4 years of age in a double-blind, randomized trial of long-chain polyunsaturated fatty acid-supplemented infant formula. *Early Hum Dev.* 2007;83(5):279-284.

159. Martin RM, Ness AR, Gunnell D, Emmett P, Davey SG, Team AS. Does breast-feeding in infancy lower blood pressure in childhood? The Avon Longitudinal Study of Parents and Children (ALSPAC). *Circulation.* 2004;109(10):1259-1266.

160. Taittonen L, Nuutinen M, Turtinen J, Uhari M. Prenatal and postnatal factors in predicting later blood pressure among children: cardiovascular risk in young Finns. *Pediatric Research.* 1996;40(4):627-632.

161. Singhal A, Cole TJ, Lucas A. Early nutrition in preterm infants and later blood pressure: two cohorts after randomised trials. *Lancet.* 2001;357(9254):413-419.

162. Forsyth J, Willatts P, Agostoni C, Bissenden J, Casaer P, Boehm G. Long chain polyunsaturated fatty acid supplementation in infant formula and blood pressure in later childhood: follow up of a randomised controlled trial. *BMJ.* 2003;326(7396):953.

163. Ravelli AC, van der Meulen JH, Osmond C, Barker DJ, Bleker OP. Infant feeding and adult glucose tolerance, lipid profile, blood pressure, and obesity. *Archives of Disease in Childhood.* 2000;82(3):248-252.

164. Parsons TJ, Power C, Manor O. Infant feeding and obesity through the lifecourse. *Arch Dis Child.* 2003;88.

165. *Indicators for Assessing Breast-Feeding Practices. WHO Document WHO/CDD/SER/91.14.* Geneva: World Health Organization; 1991.

# Trial Registration isrctn.org: ISRCTN37687716:

ISRCTN37687716 DOI 10.1186/ISRCTN37687716

PROmotion of Breastfeeding Intervention

Trial

Condition category

Pregnancy and Childbirth

Date applied

25/02/2005

Date assigned

09/09/2005

Last edited

13/02/2015

Prospective/Retrospective Retrospectively registered

Overall trial status Completed

Recruitment status No longer recruiting

# Plain English Summary

Background and study aims

The Promotion of Breastfeeding Intervention Trial (PROBIT) is a study in the Republic of Belarus involving 31 maternity hospitals and affiliated clinics across the country. The study was designed to help scientists, health care providers, and the general public understand the effects of infant feeding on child health and development.

In 1995, 16 hospitals and clinics were randomly allocated to a breastfeeding promotion intervention based on World Health Organization materials and procedures, while 15 continued breastfeeding practices in place at the time of random allocation. Mothers and babies were recruited from June 1996 to December 1997. In total, 17,046 mothers and their babies were recruited into the study. Of these, 16,492 (97%) were followed at regular intervals until the infants were 12 months of age. Detailed information was recorded at each followup visit about infant feeding, digestive and lung infections, and rashes.

When PROBIT children were six and a half years old, 13,889 (81%) were examined for height, weight, body fat, blood pressure, behaviour, dental health, intelligence quotient (IQ), asthma and allergy. At age 11 and a half years, 13,879 (81%) were again examined for height, weight, body fat, blood pressure, and also had blood tests to measure diabetes and heart disease risk factors. Currently we are seeing the children at age 16 years.

Who can participate?

Recruitment is complete. Mothers and their babies joined the study during their delivery hospital stay. Mothers could take part if they started breastfeeding, and they and their baby were healthy.

What does the study involve?

At the current visit, when the child is 16, the pediatrician:

1. Measures the child’s height, waist, blood pressure, weight and body fat.
2. Tests the child's vision by asking him/her to read letter charts. A small number of children are also testedusing an instrument that assesses whether they need glasses. 3. Examines the child’s skin for rashes.
3. Tests the child’s lung health by asking him or her to blow into a tube three to eight times to measure the capacity of the lungs.
4. Asks the child to take a computer­administered test of brain development (including memory, ability tosolve problems, attention, perception, verbal skills, information processing and motor skills).
5. Administers a questionnaire to assess other aspects of the child’s health and physical development.

What are the possible benefits and risks of participating?

The lung function assessment involves blowing hard into a tube several times. Repeated blowing may cause some people to become wheezy. The pediatrician has asthma medication on hand to relieve these symptoms if they occur. None of the other measures or tests carries any risk to the child. As a result of the examination, the pediatrician may identify previously undiagnosed eye, lung or blood pressure problems in the child, which will then be followed up appropriately.

Where is the study run from?

The study is run from the The National Research and Applied Medicine Mother and Child Centre (Minsk,

Belarus), in collaboration with the School of Social and Community Medicine, University of Bristol (Bristol,

UK), Departments of Pediatrics and of Epidemiology, Biostatistics and Occupational Health, McGill

University Faculty of Medicine (Montreal, Canada), and Harvard Medical School and Harvard Pilgrim

Health Care Institute (Boston, USA).

When is the study starting and how long is it expected to run for?

The study started in January 1995 and is expected to run until December 2015. We hope the study will extend beyond this time as we intend to look at the children’s health over many years.

Who is funding the study?

This study is supported by a grant from the Canadian Institutes of Health Research (CIHR) and the US National Institutes of Health (NIH). The study has previously been funded by the National Health Research and Development Program (NHRDP) Health Canada, European Union’s project on Early Nutrition

Programming: Long­term Efficacy and Safety Trials, the Thrasher Research Fund (USA), the United Nations Children’s Fund (UNICEF), and the European Regional Office of the World Health Organization (WHO).

Who is the main contact?

Professor Michael S Kramer

Michael.Kramer@mcgill.ca Trial website

http://www.bristol.ac.uk/social­community­medicine/projects/probit/ [http://www.bristol.ac.uk/socialcommunity­medicine/projects/probit/]

# Contact information

Type

Scientific

Primary contact

Dr Michael S. Kramer

ORCID ID

[]

## Contact details

The Montreal Children's Hospital

2300 Tupper Street

T­118

Montreal

H3H 1P3

Canada

+1 (0)514 4124400 ext. 22687

michael.kramer@mcgill.ca [<mailto:michael.kramer@mcgill.ca>]

# Additional identifiers

EudraCT number ClinicalTrials.gov number

NCT01561612

Protocol/serial number

MOP­53155

# Study information

Scientific title

Breastfeeding duration and exclusivity: impact on child health and development

Acronym

PROBIT

## Study hypothesis

Current study hypothesis as of 11/03/2009:

Experimental intervention will lead to increased exclusivity and duration of breastfeeding, and hence to improved infant and child health.

Initial information at time of registration:

Experimental intervention will lead to increased exclusivity and duration of breastfeeding, and hence to reduced infection and eczema in infancy.

As of 25/03/2009, this record has been updated to include an updated anticipated end date; the initial end date at the time of registration was 31/03/2008.

As of 09/01/2013, the following changes were made to the record:

1. The anticipated end date for this trial was updated from 01/12/2011 to 31/12/2015
2. Belarus was added to the countries of recruitment, and Canada was removed

As of 02/09/2013, the anticipated start date was changed from 01/04/2002 to 01/01/1995.

## Ethics approval

Research Ethics Board of McGill University Health Centre approved on 28/11/2001

Added 02/09/2013: Research Ethics Board of McGill University Health Centre approved on 18/06/2012

(Ref: 11­190­PED)

Study design

Randomised controlled trial

Primary study design

Interventional

Secondary study design

Randomised controlled trial

Trial setting

Hospitals

Trial type

Prevention

Patient information sheet Condition

Healthy, full­term, breastfed infants

## Intervention

Experimental group: breastfeeding promotion intervention at maternity hospitals and affiliated polyclinics

Control group: continuation of maternity hospital and polyclinic practices existing at time of randomisation

Intervention type

Other

Phase

Not Applicable

Drug names Primary outcome measures

One or more episodes of gastrointestinal infection in first 12 months of life.

## Secondary outcome measures

Current secondary outcome measures as of 09/01/2013:

1. Respiratory infections in first 12 months
2. Atopic eczema in first 12 months
3. Weight, length, and head circumference at 1, 2, 3, 6, 9, and 12 months
4. Blood pressure (BP) at age 6.5 and 9 years
5. Asthma, hay fever, atopic eczema, and allergy skin tests at age 6.5 years
6. Intelligence quotient (IQ) and behaviour at age 6.5 years
7. Oral/dental health at age 6.5 years
8. Anthropometry, lipoproteins, glucose, insulin, adiponectin, and IGF at age 11 years
9. Maternal height and weight at 6.5 and 11.5 years postpartum
10. Maternal body composition at 11.5 years postpartum
11. Maternal blood pressure at 11.5 years postpartum
12. Child metabolic syndrome at age 11.5 years
13. Eating attitudes at age 11.5 years
14. Child blood pressure at age 6.5, 11.5 and 16 years
15. Child body composition at age 11.5 and 16 years
16. Eczema, asthma, cognition, vision and lung function at age 16 years
17. Length/height and weight throughout childhood

Amended as of 11/03/2009:

8. Anthropometry, lipoproteins, glucose, insulin, adiponectin, and IGF at age 11 years

Initial information at time of registration:

1. Respiratory infections in first 12 months
2. Atopic eczema in first 12 months
3. Weight, length, and head circumference at 1, 2, 3, 6, 9, and 12 months
4. Blood pressure (BP) at age 6.5 and 9 years
5. Asthma, hay fever, atopic eczema, and allergy skin tests at age 6.5 years
6. Intelligence quotient (IQ) and behaviour at age 6.5 years
7. Oral/dental health at age 6.5 years
8. Lipids, lipoproteins, glucose, insulin, and HbA1c at age 9 years

Overall trial start date

01/01/1995

Overall trial end date

31/12/2015

Reason abandoned

# Eligibility

## Participant inclusion criteria

1. Birth weight equal and above 2500 g, either sex
2. Gestational age equal and above 37 weeks
3. Maternal intention to breastfeed

Participant type

Patient

Age group

Neonate

Gender

Both

Target number of participants

17046

## Participant exclusion criteria

1. Neonatal disease or condition contraindicating breastfeeding
2. Neonatal disease or condition making breastfeeding difficult or impossible
3. Maternal psychosis
4. Maternal human immunodeficiency virus (HIV) or active tuberculosis (TB)
5. Maternal chemotherapy or radioisotopes

Recruitment start date

01/01/1995

Recruitment end date

31/12/2015

# Locations

Countries of recruitment

Belarus

## Trial participating centre

The Montreal Children's Hospital

Montreal

H3H 1P3 Canada

# Sponsor information

Organisation

McGill University (Canada)

## Sponsor details

845 Sherbrooke Street West James Administration Bldg.

Suite 429 Montreal

H3A 2T5

Canada

Sponsor type

University/education

Website

http://www.mcgill.ca/ [http://www.mcgill.ca/]

# Funders

Funder type

Research organisation

## Funder name

Canadian Institutes of Health Research (CIHR) (Canada) ­ http://www.cihr­irsc.gc.ca [http://www.cihrirsc.gc.ca] (ref: MOP­53155)

Alternative name(s) Funding Body Type Funding Body Subtype Location Funder name

United Nations Children's Fund (UNICEF)

Alternative name(s)

Funding Body Type Funding Body Subtype Location Funder name

Thrasher Research Fund (USA)

Alternative name(s) Funding Body Type

private sector organisation

Funding Body Subtype

foundation

Location

United States of America

Funder name

National Health Research and Development Program (NHRDP) ­ Health Canada (Canada)

Alternative name(s) Funding Body Type Funding Body Subtype Location Funder name

European Union (EU)

Alternative name(s) Funding Body Type Funding Body Subtype Location Funder name

National Institutes of Health (NIH) (USA)

Alternative name(s)

NIH

Funding Body Type

government organisation

Funding Body Subtype

federal/national government

Location

United States of America

# Results and Publications

Publication and dissemination plan

Not provided at time of registration

Intention to publish date Participant level data

Not provided at time of registration

## Results ­ basic reporting Publication summary

1. 2007 results in: http://www.ncbi.nlm.nih.gov/pubmed/17855282

[http://www.ncbi.nlm.nih.gov/pubmed/17855282]

1. 2007 results in: http://www.ncbi.nlm.nih.gov/pubmed/18065591 [http://www.ncbi.nlm.nih.gov/pubmed/18065591]
2. 2008 results in: http://www.ncbi.nlm.nih.gov/pubmed/18310164

[http://www.ncbi.nlm.nih.gov/pubmed/18310164]

1. 2008 results in: http://www.ncbi.nlm.nih.gov/pubmed/18458209

[http://www.ncbi.nlm.nih.gov/pubmed/18458209]

1. 2011 results in: http://www.ncbi.nlm.nih.gov/pubmed/21633072

[http://www.ncbi.nlm.nih.gov/pubmed/21633072]

1. 2013 results in: http://www.ncbi.nlm.nih.gov/pubmed/23483175

[http://www.ncbi.nlm.nih.gov/pubmed/23483175]

1. 2013 results in: http://www.ncbi.nlm.nih.gov/pubmed/23776123

[http://www.ncbi.nlm.nih.gov/pubmed/23776123]

1. 2013 results in: http://www.ncbi.nlm.nih.gov/pubmed/24300437

[http://www.ncbi.nlm.nih.gov/pubmed/24300437]

1. 2014 results in: http://www.ncbi.nlm.nih.gov/pubmed/24706729

[http://www.ncbi.nlm.nih.gov/pubmed/24706729]

1. 2014 results in: http://www.ncbi.nlm.nih.gov/pubmed/24787489

[http://www.ncbi.nlm.nih.gov/pubmed/24787489]

1. 2014 cohort profile in: http://www.ncbi.nlm.nih.gov/pubmed/23471837 [http://www.ncbi.nlm.nih.gov/pubmed/23471837]

## Publication citations

1. Results

Kramer MS, Matush L, Vanilovich I, Platt R, Bogdanovich N, Sevkovskaya Z, Dzikovich I, Shishko G, Mazer B, , Effect of prolonged and exclusive breast feeding on risk of allergy and asthma: cluster randomised trial., BMJ, 2007, 335, 7624, 815, doi: 10.1136/bmj.39304.464016.AE.

PubMed Abstract [http://www.ncbi.nlm.nih.gov/pubmed/17855282] Publisher Full Text [http://doi.org/10.1136/bmj.39304.464016.AE]

1. Results

Kramer MS, Matush L, Vanilovich I, Platt RW, Bogdanovich N, Sevkovskaya Z, Dzikovich I, Shishko G, Collet JP, Martin RM, Davey Smith G, Gillman MW, Chalmers B, Hodnett E, Shapiro S, , Effects of prolonged and exclusive breastfeeding on child height, weight, adiposity, and blood pressure at age 6.5 y: evidence from a large randomized trial., Am. J. Clin. Nutr., 2007, 86, 6, 1717­1721.

PubMed Abstract [http://www.ncbi.nlm.nih.gov/pubmed/18065591]

1. Results

Kramer MS, Fombonne E, Igumnov S, Vanilovich I, Matush L, Mironova E, Bogdanovich N, Tremblay RE, Chalmers B, Zhang X, Platt RW, , Effects of prolonged and exclusive breastfeeding on child behavior and maternal adjustment: evidence from a large, randomized trial., Pediatrics, 2008, 121, 3, e435­40, doi: 10.1542/peds.2007­1248.

PubMed Abstract [http://www.ncbi.nlm.nih.gov/pubmed/18310164] Publisher Full Text [http://doi.org/10.1542/peds.2007­1248]

1. Results

Kramer MS, Aboud F, Mironova E, Vanilovich I, Platt RW, Matush L, Igumnov S, Fombonne E,

Bogdanovich N, Ducruet T, Collet JP, Chalmers B, Hodnett E, Davidovsky S, Skugarevsky O, Trofimovich O, Kozlova L, Shapiro S, , Breastfeeding and child cognitive development: new evidence from a large randomized trial., Arch. Gen. Psychiatry, 2008, 65, 5, 578­584, doi:

10.1001/archpsyc.65.5.578.

PubMed Abstract [http://www.ncbi.nlm.nih.gov/pubmed/18458209] Publisher Full Text [http://doi.org/10.1001/archpsyc.65.5.578]

1. Results

Tilling K, Davies NM, Nicoli E, Ben­Shlomo Y, Kramer MS, Patel R, Oken E, Martin RM, Associations of growth trajectories in infancy and early childhood with later childhood outcomes., Am. J. Clin. Nutr., 2011, 94, 6 Suppl, 1808S­1813S, doi: 10.3945/ajcn.110.001644.

PubMed Abstract [http://www.ncbi.nlm.nih.gov/pubmed/21633072] Publisher Full Text [http://doi.org/10.3945/ajcn.110.001644]

1. Results

Martin RM, Patel R, Kramer MS, Guthrie L, Vilchuck K, Bogdanovich N, Sergeichick N, Gusina N, Foo Y, Palmer T, Rifas­Shiman SL, Gillman MW, Smith GD, Oken E, Effects of promoting longerterm and exclusive breastfeeding on adiposity and insulin­like growth factor­I at age 11.5 years: a randomized trial., JAMA, 2013, 309, 10, 1005­1013, doi: 10.1001/jama.2013.167.

PubMed Abstract [http://www.ncbi.nlm.nih.gov/pubmed/23483175] Publisher Full Text [http://doi.org/10.1001/jama.2013.167]

1. Results

Smithers LG, Lynch JW, Yang S, Dahhou M, Kramer MS, Impact of neonatal growth on IQ and behavior at early school age., Pediatrics, 2013, 132, 1, e53­60, doi: 10.1542/peds.2012­3497.

PubMed Abstract [http://www.ncbi.nlm.nih.gov/pubmed/23776123] Publisher Full Text [http://doi.org/10.1542/peds.2012­3497]

1. Results

Martin RM, Patel R, Kramer MS, Vilchuck K, Bogdanovich N, Sergeichick N, Gusina N, Foo Y, Palmer T, Thompson J, Gillman MW, Smith GD, Oken E, Effects of promoting longer­term and exclusive breastfeeding on cardiometabolic risk factors at age 11.5 years: a cluster­randomized, controlled trial., Circulation, 2014, 129, 3, 321­329, doi: 10.1161/CIRCULATIONAHA.113.005160.

PubMed Abstract [http://www.ncbi.nlm.nih.gov/pubmed/24300437]

Publisher Full Text [http://doi.org/10.1161/CIRCULATIONAHA.113.005160]

1. Results

Skugarevsky O, Wade KH, Richmond RC, Martin RM, Tilling K, Patel R, Vilchuck K, Bogdanovich N, Sergeichick N, Davey Smith G, Gillman MW, Oken E, Kramer MS, Effects of promoting longer­term and exclusive breastfeeding on childhood eating attitudes: a cluster­randomized trial., Int J Epidemiol, 2014, 43, 4, 1263­1271, doi: 10.1093/ije/dyu072.

PubMed Abstract [http://www.ncbi.nlm.nih.gov/pubmed/24706729] Publisher Full Text [http://doi.org/10.1093/ije/dyu072]

1. Results

Kramer MS, Martin RM, Bogdanovich N, Vilchuk K, Dahhou M, Oken E, Is restricted fetal growth associated with later adiposity? Observational analysis of a randomized trial., Am. J. Clin. Nutr., 2014, 100, 1, 176­181, doi: 10.3945/ajcn.113.079590.

PubMed Abstract [http://www.ncbi.nlm.nih.gov/pubmed/24787489] Publisher Full Text [http://doi.org/10.3945/ajcn.113.079590]

1. Cohort profile

Patel R, Oken E, Bogdanovich N, Matush L, Sevkovskaya Z, Chalmers B, Hodnett ED, Vilchuck K, Kramer MS, Martin RM, Cohort profile: The promotion of breastfeeding intervention trial (PROBIT), Int J Epidemiol, 2014, 43, 3, 679­690, doi: 10.1093/ije/dyt003.

PubMed Abstract [http://www.ncbi.nlm.nih.gov/pubmed/23471837] Publisher Full Text [http://doi.org/10.1093/ije/dyt003]

# Additional files Editorial Notes

**Trial Registration clinicaltrials.gov: NCT01561612**

IGF from dried bloodspots

child height [ Time Frame: throughout childhood ] Research measures of length/height

adiponectin [ Time Frame: age 11.5 years ] adiponectin from dried bloodspot

Apo A1 [ Time Frame: Child age 11.5 years ] Apo A1 in dried bloodspots

glucose [ Time Frame: child age 11.5 years ] fingerstick glucose measured by glucometer

insulin [ Time Frame: child age 11.5 years ] insulin measured on dried blood spots

Apo B [ Time Frame: Child age 11.5 years ]

Apo B measured in dried blood spots

Child blood pressure [ Time Frame: throughout childhood ]

Research measures of blood pressure at ages 6.5, 11.5 and 16 years

Maternal blood pressure [ Time Frame: 11.5 years postpartum ]

Research measure of maternal blood pressure

Child metabolic syndrome [ Time Frame: Age 11.5 years ]

Child growth [ Time Frame: Throughout childhood ]

Growth in weight, length, weight for length, BMI, and other measures

Respiratory tract infections [ Time Frame: to age 12 months and throughout childhood ]

Number of respiratory infections, from review of medical record

Atopic Eczema [ Time Frame: Throughout childhood ]

Eczema from review of medical record, parent report,and direct examination

Atopy [ Time Frame: Age 6.5 years ] Skinprick tests for allergy

Eating Attitudes [ Time Frame: Age 11.5 years ]

Children's Eating Attitudes Test (ChEAT) Questionnaire

Enrollment: 17046

Study Start Date: June 1996

Primary Completion Date: December 1998 (Final data collection date for primary outcome measure)

| Arms | Assigned Interventions |
| --- | --- |
| Experimental: Intervention  Breastfeeding promotion according to World Health  Organization's Baby Friendly Hospital Initiative | Behavioral: Breastfeeding promotion and support  Breastfeeding promotion and support according to the World Health  Organization's Baby Friendly Hospital Initiative |
| No Intervention: Control  Usual care |  |

Eligibility

Ages Eligible for Study: Child, Adult, Senior

Sexes Eligible for Study: All

Accepts Healthy Volunteers: No

Criteria

Inclusion Criteria:

Birth at one of 34 Maternity Hospitals in Republic of Belarus

Breastfeeding initiated at birth, with no contraindications to breastfeeding

Apgar score >=5 at 5 minutes

Full term gestation

Birth weight > 2500g

Contacts and Locations

Choosing to participate in a study is an important personal decision. Talk with your doctor and family members or friends about deciding to join a study. To learn more about this study, you or your doctor may contact the study research staff using the Contacts provided below. For general information, see Learn About Clinical Studies.

Please refer to this study by its ClinicalTrials.gov identifier: NCT01561612

Locations

Belarus

Maternal and Child Health Center

Minsk, Belarus

Sponsors and Collaborators

Harvard Pilgrim Health Care

Eunice Kennedy Shriver National Institute of Child Health and Human Development (NICHD)

Canadian Institutes of Health Research (CIHR)

European Union EarlyNutrition program University of Bristol

Investigators

Principal Investigator: Emily Oken, MD Harvard Pilgrim Health Care Principal Investigator: Richard M Martin, MD University of Bristol

More Information

Additional Information:

Related Info
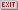


Publications:

Kramer MS, Chalmers B, Hodnett ED, Sevkovskaya Z, Dzikovich I, Shapiro S, Collet JP, Vanilovich I, Mezen I, Ducruet T, Shishko G, Zubovich V, Mknuik D, Gluchanina E, Dombrovsky V, Ustinovitch A, Ko T, Bogdanovich N, Ovchinikova L, Helsing E. Promotion of breastfeeding intervention trial (PROBIT): a cluster­randomized trial in the Republic of Belarus. Design, follow­up, and data validation. Adv Exp Med Biol. 2000;478:327­45.

Kramer MS, Chalmers B, Hodnett ED, Sevkovskaya Z, Dzikovich I, Shapiro S, Collet JP, Vanilovich I, Mezen I, Ducruet T, Shishko G, Zubovich V, Mknuik D, Gluchanina E, Dombrovskiy V, Ustinovitch A, Kot T, Bogdanovich N, Ovchinikova L, Helsing E; PROBIT Study Group (Promotion of Breastfeeding Intervention Trial).. Promotion of Breastfeeding Intervention Trial (PROBIT): a randomized trial in the Republic of Belarus. JAMA. 2001 Jan 24­31;285(4):413­20.


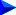

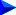


Kramer MS, Guo T, Platt RW, Shapiro S, Collet JP, Chalmers B, Hodnett E, Sevkovskaya Z, Dzikovich I, Vanilovich I; PROBIT Study Group.. Breastfeeding and infant growth: biology or bias? Pediatrics. 2002 Aug;110(2 Pt 1):343­7.

Kramer MS, Guo T, Platt RW, Sevkovskaya Z, Dzikovich I, Collet JP, Shapiro S, Chalmers B, Hodnett E, Vanilovich I, Mezen I, Ducruet T, Shishko

G, Bogdanovich N. Infant growth and health outcomes associated with 3 compared with 6 mo of exclusive breastfeeding. Am J Clin Nutr. 2003 Aug;78(2):291­5.

Kramer MS, Guo T, Platt RW, Sevkovskaya Z, Dzikovich I, Collet JP, Shapiro S, Chalmers B, Hodnett E, Vanilovich I, Mezen I, Ducruet T, Shishko G, Bogdanovich N. Does previous infection protect against atopic eczema and recurrent wheeze in infancy? Clin Exp Allergy. 2004 May;34(5):753­6.

Kramer MS, Kakuma R. The optimal duration of exclusive breastfeeding: a systematic review. Adv Exp Med Biol. 2004;554:63­77. Review.

Kramer MS, Vanilovich I, Matush L, Bogdanovich N, Zhang X, Shishko G, Muller­Bolla M, Platt RW. The effect of prolonged and exclusive breastfeeding on dental caries in early school­age children. New evidence from a large randomized trial. Caries Res. 2007;41(6):484­8.

Kramer MS, Matush L, Vanilovich I, Platt R, Bogdanovich N, Sevkovskaya Z, Dzikovich I, Shishko G, Mazer B; Promotion of Breastfeeding Intervention Trial (PROBIT) Study Group.. Effect of prolonged and exclusive breast feeding on risk of allergy and asthma: cluster randomised trial. BMJ. 2007 Oct 20;335(7624):815.

Kramer MS. Allergy after breast feeding. Testing hypotheses: reply. BMJ. 2007 Nov 24;335(7629):1061­2.

Kramer MS, Matush L, Vanilovich I, Platt RW, Bogdanovich N, Sevkovskaya Z, Dzikovich I, Shishko G, Collet JP, Martin RM, Davey Smith G, Gillman MW, Chalmers B, Hodnett E, Shapiro S; PROBIT Study Group.. Effects of prolonged and exclusive breastfeeding on child height, weight, adiposity, and blood pressure at age 6.5 y: evidence from a large randomized trial. Am J Clin Nutr. 2007 Dec;86(6):1717­21.

Kramer MS, Fombonne E, Igumnov S, Vanilovich I, Matush L, Mironova E, Bogdanovich N, Tremblay RE, Chalmers B, Zhang X, Platt RW; Promotion of Breastfeeding Intervention Trial (PROBIT) Study Group.. Effects of prolonged and exclusive breastfeeding on child behavior and maternal adjustment: evidence from a large, randomized trial. Pediatrics. 2008 Mar;121(3):e435­40. doi: 10.1542/peds.2007­1248.

Kramer MS, Matush L, Vanilovich I, Platt RW, Bogdanovich N, Sevkovskaya Z, Dzikovich I, Shishko G, Collet JP, Martin RM, Smith GD, Gillman MW, Chalmers B, Hodnett E, Shapiro S. A randomized breast­feeding promotion intervention did not reduce child obesity in Belarus. J Nutr. 2009 Feb;139(2):417S­21S. doi: 10.3945/jn.108.097675.

Kramer MS. Methodological challenges in studying long­term effects of breast­feeding. Adv Exp Med Biol. 2009;639:121­33. doi: 10.1007/978­14020­8749­3_10. Review.

Kramer MS, Matush L, Bogdanovich N, Dahhou M, Platt RW, Mazer B. The low prevalence of allergic disease in Eastern Europe: are risk factors consistent with the hygiene hypothesis? Clin Exp Allergy. 2009 May;39(5):708­16. doi: 10.1111/j.1365­2222.2009.03205.x.

Kramer MS, Martin RM, Sterne JA, Shapiro S, Dahhou M, Platt RW. The double jeopardy of clustered measurement and cluster randomisation. BMJ. 2009 Aug 21;339:b2900. doi: 10.1136/bmj.b2900.

Kramer MS, Matush L, Bogdanovich N, Aboud F, Mazer B, Fombonne E, Collet JP, Hodnett E, Mironova E, Igumnov S, Chalmers B, Dahhou M, Platt RW. Health and development outcomes in 6.5­y­old children breastfed exclusively for 3 or 6 mo. Am J Clin Nutr. 2009 Oct;90(4):1070­4. doi: 10.3945/ajcn.2009.28021.

Yang S, Platt RW, Kramer MS. Variation in child cognitive ability by week of gestation among healthy term births. Am J Epidemiol. 2010 Feb 15;171(4):399­406. doi: 10.1093/aje/kwp413.

Kramer MS. Breastfeeding, complementary (solid) foods, and long­term risk of obesity. Am J Clin Nutr. 2010 Mar;91(3):500­1. doi:

10.3945/ajcn.2010.29199.

Kramer MS. Long­term behavioral consequences of infant feeding: the limits of observational studies. J Pediatr. 2010 Apr;156(4):523­4. doi:

10.1016/j.jpeds.2009.12.002.

Kramer MS. "Breast is best": The evidence. Early Hum Dev. 2010 Nov;86(11):729­32. doi: 10.1016/j.earlhumdev.2010.08.005.

Martin RM, Kramer MS, Dahhou M, Platt RW, Patel R, Bogdanovich N, Matush L, Davey Smith G; Promotion of Breastfeeding Intervention Trial (PROBIT) Study Group.. Do gastrointestinal tract infections in infancy increase blood pressure in childhood? A cohort study. J Epidemiol Community Health. 2010 Dec;64(12):1068­73. doi: 10.1136/jech.2009.090894.

Patel R, Martin RM, Kramer MS, Oken E, Bogdanovich N, Matush L, Smith GD, Lawlor DA. Familial associations of adiposity: findings from a cross­sectional study of 12,181 parental­offspring trios from Belarus. PLoS One. 2011 Jan 27;6(1):e14607. doi: 10.1371/journal.pone.0014607.

Patel R, Lawlor DA, Kramer MS, Smith GD, Bogdanovich N, Matush L, Martin RM. Socio­economic position and adiposity among children and their parents in the Republic of Belarus. Eur J Public Health. 2011 Apr;21(2):158­65. doi: 10.1093/eurpub/ckq041.

Kramer MS, Moodie EE, Dahhou M, Platt RW. Breastfeeding and infant size: evidence of reverse causality. Am J Epidemiol. 2011 May 1;173(9):978­83. doi: 10.1093/aje/kwq495.

Yang S, Fombonne E, Kramer MS. Duration of gestation, size at birth and later childhood behaviour. Paediatr Perinat Epidemiol. 2011 Jul;25(4):377­87. doi: 10.1111/j.1365­3016.2011.01193.x.

Patel R, Lawlor DA, Kramer MS, Davey Smith G, Bogdanovich N, Matush L, Martin RM. Socioeconomic inequalities in height, leg length and trunk length among children aged 6.5 years and their parents from the Republic of Belarus: evidence from the Promotion of Breastfeeding Intervention Trial (PROBIT). Ann Hum Biol. 2011 Sep;38(5):592­602. doi: 10.3109/03014460.2011.577752.

Tilling K, Davies N, Windmeijer F, Kramer MS, Bogdanovich N, Matush L, Patel R, Smith GD, Ben­Shlomo Y, Martin RM; Promotion of

Breastfeeding Intervention Trial (PROBIT) study group.. Is infant weight associated with childhood blood pressure? Analysis of the Promotion of Breastfeeding Intervention Trial (PROBIT) cohort. Int J Epidemiol. 2011 Oct;40(5):1227­37. doi: 10.1093/ije/dyr119.

Yang S, Tilling K, Martin R, Davies N, Ben­Shlomo Y, Kramer MS. Pre­natal and post­natal growth trajectories and childhood cognitive ability and mental health. Int J Epidemiol. 2011 Oct;40(5):1215­26. doi: 10.1093/ije/dyr094.

Kramer MS, Fombonne E, Matush L, Bogdanovich N, Dahhou M, Platt RW. Long­term behavioural consequences of infant feeding: the limits of observational studies. Paediatr Perinat Epidemiol. 2011 Nov;25(6):500­6. doi: 10.1111/j.1365­3016.2011.01211.x.

Tilling K, Davies NM, Nicoli E, Ben­Shlomo Y, Kramer MS, Patel R, Oken E, Martin RM. Associations of growth trajectories in infancy and early childhood with later childhood outcomes. Am J Clin Nutr. 2011 Dec;94(6 Suppl):1808S­1813S. doi: 10.3945/ajcn.110.001644.

Kramer MS. Breastfeeding and allergy: the evidence. Ann Nutr Metab. 2011;59 Suppl 1:20­6. doi: 10.1159/000334148. Review.

Yang S, Kramer MS. Paternal alcohol consumption, family transition and child development in a former Soviet country. Int J Epidemiol. 2012 Aug;41(4):1086­96. doi: 10.1093/ije/dys071.

Kramer MS, Moodie EE, Platt RW. Infant feeding and growth: can we answer the causal question? Epidemiology. 2012 Nov;23(6):790­4. doi:

10.1097/EDE.0b013e31826cc0e9.

Martin RM, Patel R, Zinovik A, Kramer MS, Oken E, Vilchuck K, Bogdanovich N, Sergeichick N, Gunnarsson R, Grufman L, Foo Y, Gusina N.

Filter paper blood spot enzyme linked immunoassay for insulin and application in the evaluation of determinants of child insulin resistance. PLoS One. 2012;7(10):e46752. doi: 10.1371/journal.pone.0046752.

Patel R, Oken E, Bogdanovich N, Matush L, Sevkovskaya Z, Chalmers B, Hodnett ED, Vilchuck K, Kramer MS, Martin RM. Cohort profile: The promotion of breastfeeding intervention trial (PROBIT). Int J Epidemiol. 2014 Jun;43(3):679­90. doi: 10.1093/ije/dyt003.

Martin RM, Patel R, Kramer MS, Guthrie L, Vilchuck K, Bogdanovich N, Sergeichick N, Gusina N, Foo Y, Palmer T, Rifas­Shiman SL, Gillman MW, Smith GD, Oken E. Effects of promoting longer­term and exclusive breastfeeding on adiposity and insulin­like growth factor­I at age 11.5 years: a randomized trial. JAMA. 2013 Mar 13;309(10):1005­13. doi: 10.1001/jama.2013.167.

Anderson EL, Fraser A, Martin RM, Kramer MS, Oken E, Patel R, Tilling K; PROBIT Study.. Associations of postnatal growth with asthma and atopy: the PROBIT Study. Pediatr Allergy Immunol. 2013 Mar;24(2):122­30. doi: 10.1111/pai.12049.

Publications automatically indexed to this study by ClinicalTrials.gov Identifier (NCT Number):

Wade KH, Kramer MS, Oken E, Timpson NJ, Skugarevsky O, Patel R, Bogdanovich N, Vilchuck K, Davey Smith G, Thompson J, Martin RM. Prospective associations between problematic eating attitudes in midchildhood and the future onset of adolescent obesity and high blood pressure. Am J Clin Nutr. 2017 Feb;105(2):306­312. doi: 10.3945/ajcn.116.141697.

Patel R, Tilling K, Lawlor DA, Howe LD, Bogdanovich N, Matush L, Nicoli E, Kramer MS, Martin RM. Socioeconomic differences in childhood length/height trajectories in a middle­income country: a cohort study. BMC Public Health. 2014 Sep 8;14:932. doi: 10.1186/1471­2458­14­932.

Martin RM, Patel R, Kramer MS, Vilchuck K, Bogdanovich N, Sergeichick N, Gusina N, Foo Y, Palmer T, Thompson J, Gillman MW, Smith GD, Oken E. Effects of promoting longer­term and exclusive breastfeeding on cardiometabolic risk factors at age 11.5 years: a cluster­randomized, controlled trial. Circulation. 2014 Jan 21;129(3):321­9. doi: 10.1161/CIRCULATIONAHA.113.005160.

Oken E, Patel R, Guthrie LB, Vilchuck K, Bogdanovich N, Sergeichick N, Palmer TM, Kramer MS, Martin RM. Effects of an intervention to promote breastfeeding on maternal adiposity and blood pressure at 11.5 y postpartum: results from the Promotion of Breastfeeding Intervention Trial, a cluster­randomized controlled trial. Am J Clin Nutr. 2013 Oct;98(4):1048­56. doi: 10.3945/ajcn.113.065300.

Responsible Party: Emily Oken, Associate Professor, Harvard Pilgrim Health Care

ClinicalTrials.gov Identifier: NCT01561612 History of Changes

Other Study ID Numbers: 190250 R01HD050758 FOOD­DT­2005­007036 G0600705 K24HD069408 MOP­53155 Study First Received: March 20, 2012

Last Updated: October 9, 2013

Keywords provided by Harvard Pilgrim Health Care:

Breastfeeding Cognitive Development

Child Health Behavior

Obesity Eczema

Blood Pressure Lung Function

Asthma Spirometry

ClinicalTrials.gov processed this record on February 10, 2017
