# Supplementary material for: Breastfeeding during infancy and neurocognitive function in adolescence: 16-year follow-up of the PROBIT cluster-randomized trial
Source: PLoS Med. 2018 Apr 20;15(4):e1002554. doi: 10.1371/journal.pmed.1002554 (PMC5909901; doi:10.1371/journal.pmed.1002554)
Supplement: S3 Text — (DOC) [file pmed.1002554.s006.doc]

**Description of 10 sub-tests and scoring of 7 index scores of specific cognitive domains in NeuroTrax Tests**

Computerized sub-tests

Verbal Memory: Ten pairs of words are presented, followed by a recognition test in which one member (the target) of a previously presented pair appears together with a list of four candidates for the other member of the pair. Participants must indicate which word of the four alternatives was paired with the target when presented previously.

Non-Verbal Memory: Eight pictures of simple geometric objects are presented, followed by a recognition test in which four versions of each object are presented, each oriented in a different direction. Participants are required to remember the orientations of the originally presented objects.

Go-NoGo test: A series of large colored stimuli are presented at pseudo-random intervals. Participants are instructed to respond as quickly as possible by pressing a mouse button if the color of the stimulus is any color except red, for which no response is to be made.

Stroop test: The Stroop is a well-established test of response inhibition (MacLeod, 1991^1^). The NeuroTrax Stroop test consists of three phases. Participants are presented with a pair of large colored squares, one on the left and the other on the right side of the screen. In each phase, participants are instructed to choose as quickly as possible which of the two squares is a particular color by pressing either the left or right mouse button, depending upon which of the two squares is the correct color. First, participants are presented with a general word in colored letters. In the next phase, participants are presented with a word that names a color in white letters. In the final phase (the Stroop phase), participants are presented with a word that names a color, but the letters of the word are in a color other than that named by the word. The instructions for the final phase are to choose the color of the letters, and not the color named by the word.

Verbal Function: Pictures of common objects of low and high familiarity are presented. Participants are instructed to select the name of the picture from four choices. In a related test, participants are instructed to select the word that best rhymes with the name of the picture.

Problem Solving: Pictorial puzzles of gradually increasing difficulty are presented. Each puzzle consists of a 2x2 array containing three black-and-white line drawings and missing element. Participants must choose the best fit for the fourth (missing) element of the puzzle from among six possible alternatives.

Visual Spatial Processing: Computer-generated scenes containing a red pillar are presented. Participants are instructed to imagine viewing the scene from the vantage point of the red pillar. Four alternative views of the scene are presented as choices.

Staged Information Processing test: This test comprises three levels of information processing load: single digits, two-digit arithmetic problems (e.g., 5-1), and three-digit arithmetic problems (e.g., 3+2-1). For each of the three levels, stimuli are presented at three different fixed rates, incrementally increasing as testing continues. Participants are instructed to respond as quickly as possible by pressing the left mouse button if the digit or result is less than or equal to 4 and the right mouse button if it is greater than 4.

Finger Tapping: Participants are instructed to tap on the mouse button for 12 seconds. This task is repeated twice for the right and left hands.

Catch Game: Participants must “catch” a rectangular white object falling vertically from the top of the screen before it reaches the bottom of the screen. Mouse button presses move a rectangular green “paddle” horizontally so that it can be positioned directly in the path of the falling object. The test requires hand-eye coordination, scanning and rapid responses.

Index Scores

Memory: Mean accuracies for learning and delayed recognition phases of Verbal and Non-Verbal Memory tests

Executive function: Composite scores (accuracy divided by reaction time) for Stroop test and Go-NoGo test, mean weighted accuracy for Catch game

Visual-spatial: Mean accuracy for Visual Spatial Processing test

Verbal function: Weighted accuracy for verbal rhyming test

Attention: Mean reaction times for the Go-NoGo test second phase of the Stroop test, mean reaction time for a low-load stage of Staged Information Processing test, mean standard deviation of reaction time for the Go-NoGo test, mean accuracy for a medium-load stage of Information Processing test

Information processing speed: Composite scores (accuracy divided by reaction time) for various low- and medium-load stages of the Staged Information Processing test

Motor skills: Mean time until first move for Catch Game, mean inter-tap interval and standard deviation of inter-tap interval for Finger Tapping test

| The table below shows how each cognitive domain index is created based on the 10 sub-tests.    Table. NeuroTrax Test Scores Contributing to Domain Index Scores | |
| --- | --- |
| Domain Index Score | Test Score |
| Memory | Verbal Memory: Total Accuracy |
|  | Delayed Verbal Memory: Accuracy |
|  | Non-Verbal Memory: Total Accuracy |
|  | Delayed Non-Verbal Memory: Accuracy |
| Executive Function | Go-NoGo: Composite Score |
|  | Stroop Interference: Composite Score, Level 3 |
|  | Catch Game: Total Score |
| Visual Spatial | Visual Spatial Processing: Accuracy |
| Verbal Function | Verbal Function: Rhyming, Accuracy |
| Attention | Go-NoGo: Rsp Time |
|  | Go-NoGo: Rsp Time Std Dev |
|  | Stroop Interference: Rsp Time, Level 2 |
|  | Staged Info Proc: Rsp Time, Level 1.2 |
|  | Staged Info Proc: Accuracy, Level 2.3 |
| Information Processing Speed | Staged Info Proc: Composite Score, Level 1.1 |
|  | Staged Info Proc: Composite Score, Level 1.3 |
|  | Staged Info Proc: Composite Score, Level 2.1 |
|  | Staged Info Proc: Composite Score, Level 2.2 |
| Motor Skills | Finger Tapping: Inter-Tap Interval |
|  | Finger Tapping: Tap Interval Std Dev |
|  | Catch Game: Time to Make 1st Move |

Composite Score = (Accuracy/Rsp Time)*100
